# *Atf3* mutant mice show reduced axon regeneration and impaired regeneration-associated gene induction after peripheral nerve injury

**DOI:** 10.1098/rsob.160091

**Published:** 2016-08-31

**Authors:** Manuel Gey, Renate Wanner, Corinna Schilling, Maria T. Pedro, Daniela Sinske, Bernd Knöll

**Affiliations:** 1 Institute of Physiological Chemistry, Ulm University, Albert-Einstein-Allee 11, 89081 Ulm, Germany; 2 Department of Neurosurgery, Bezirkskrankenhaus Günzburg, Ulm University, 89081 Ulm, Germany

**Keywords:** ATF3, axon regeneration, facial nerve, neuropeptide, RAG

## Abstract

Axon injury in the peripheral nervous system (PNS) induces a regeneration-associated gene (RAG) response. *Atf3* (activating transcription factor 3) is such a RAG and ATF3's transcriptional activity might induce ‘effector’ RAGs (e.g. small proline rich protein *1a* (*Sprr1a*)*, Galanin* (*Gal*)*,* growth-associated protein 43 (*Gap43*)) facilitating peripheral axon regeneration. We provide a first analysis of *Atf3* mouse mutants in peripheral nerve regeneration. In *Atf3* mutant mice, facial nerve regeneration and neurite outgrowth of adult ATF3-deficient primary dorsal root ganglia neurons was decreased. Using genome-wide transcriptomics, we identified a neuropeptide-encoding RAG cluster (vasoactive intestinal peptide (*Vip*)*, Ngf, Grp, Gal, Pacap*) regulated by ATF3. Exogenous administration of neuropeptides enhanced neurite growth of *Atf3* mutant mice suggesting that these molecules might be effector RAGs of ATF3's pro-regenerative function. In addition to the induction of growth-promoting molecules, we present data that ATF3 suppresses growth-inhibiting molecules such as chemokine (C-C motif) ligand 2. In summary, we show a pro-regenerative ATF3 function during PNS nerve regeneration involving transcriptional activation of a neuropeptide-encoding RAG cluster. ATF3 is a general injury-inducible factor, therefore ATF3-mediated mechanisms identified herein might apply to other cell and injury types.

## 1. Background

Upon nerve injury, the axon regeneration success varies in the adult mammalian nervous system. In the central nervous system (CNS), axon regeneration is very limited, whereas severed axons of peripheral nervous system (PNS) neurons regenerate to some extent [1–5]. One factor thought to account for this difference is the potential of PNS neurons to elicit a rapid regeneration-associated gene (RAG) response upon axotomy [5–7]. A typical RAG response encompasses several hundred genes and may vary slightly depending on the injured neuron population [8]. Nevertheless, a prototypical RAG response consists of transcription factor (TF)-encoding and ‘effector’ RAGs [2,4,6,9]. Such RAG-encoded TFs include STAT3 [10], ATF3 [11,12], c-Jun [13], Sox11 [14], Smad1 [15] and p53 [16]. Subsequently, transcriptional activity of these TF RAGs contributes to a second gene transcription wave resulting in expression of effector RAGs. Such effector RAGs encode for genes involved in cell adhesion (e.g. CD44, integrin subunits), neuropeptide signalling (e.g. *Galanin, Vip, Npy, Pacap*) and cytoskeletal modulation [17]. Subsequently, protein products of these effector RAGs can directly facilitate PNS axon regeneration, e.g. through direct axon growth stimulation [5].

In this study, we analysed ATF3, a RAG-encoded TF of the ATF/cAMP response element-binding protein (CREB) family [18,19]. In brain homeostasis, ATF3 expression is nearly undetectable, whereas pathological stimuli including axotomy, stress and epileptic seizures induce rapid ATF3 expression [18,19]. ATF3 exerts both transcriptional repression and activation of target genes including *Hsp27, Sprr1a* and *c-Jun* [18,19]. ATF3 interacts with partner proteins, e.g. c-Jun, to regulate further target genes including the CCL chemokine *Ccl2* [20–22]. Chemokine (C-C motif) ligand 2 (CCL2) is involved in neuron–immune cell interactions during axon regeneration and neuronal degeneration [23–29]. Recently, ATF3 was identified as a core hub present in a RAG network after PNS injury [8]. So far, *Atf3* mutant mice have not been investigated in axonal regeneration; however, ATF3 overexpression revealed pro-regenerative and neuroprotective functions. This includes stimulation of primary dorsal root ganglia (DRG) neurite growth and regeneration of peripheral and central DRG branches *in vivo* [8,11,12,30]. In general, ATF3 emerges as a neuroprotective factor. In mouse ALS [31] and epilepsy [32] models, ATF3 overexpression resulted in reduced neurodegeneration. This might be accomplished by ATF3's impact on dendrites, e.g. by providing protection against NMDA-exerted dendrotoxicity [33,34]. In addition, dendritic spine impairments observed in tuberous sclerosis mouse models depend on ATF3 function [35].

Herein, we provide a first analysis of constitutive *Atf3* null mouse mutation in PNS axon regeneration employing facial nerve axotomy. The facial nerve connects facial motoneurons (FMN) residing in the facial nucleus (FN) of the brainstem with facial muscles involved in stirring e.g. whisker and eyelid movement [36]. Facial nerve injury triggers a robust RAG response upon injury including ATF3 upregulation [37–42]. We observed reduced facial nerve regeneration in *Atf3* mutant mice *in vivo* and decreased neurite outgrowth on primary DRG neurons. This corresponded with a reduced RAG response in *Atf3* mutant mice resulting in blunted expression of a distinct neuropeptide encoding cluster (*Vip, Galanin, Grp, Ngf*) as well as specific other genes (e.g. *Sprr2j,* wingless-type MMTV integration site family, member 2B (*Wnt2b*)*, Ccl2*). The addition of recombinant neuropeptides rescued neurite growth inflicted by ATF3 deficiency. Thus, some of these neuropeptide-encoding genes might operate as effector RAGs transmitting ATF3's pro-regenerative function *in vivo*.

## 2. Material and methods

### 2.1. *Atf3* mutant mice

**Figure 1.**
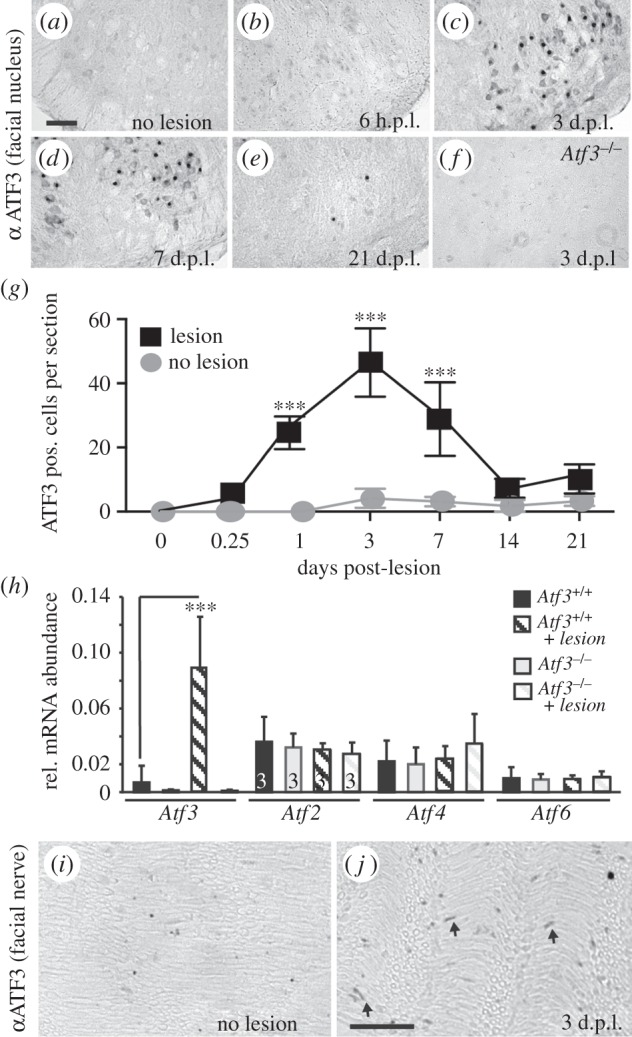
Temporal sequence ATF3 induction in injured facial motoneurons. (*a–f*) FN of wt (*a–e*) and *Atf3* mutant (*f*) animals were stained for ATF3 expression before (*a*) or several days post-lesion (d.p.l.; *b–f*). ATF3 is not present in uninjured FMNs (*a*) or only weakly at 6 h after lesion (*b*). By contrast, at 3 (*c*) and 7 (*d*) d.p.l., ATF3 was localized to FMNs. After three weeks of lesion, there was almost no ATF3 expression (*e*). In *Atf3* mutant mice, no ATF3 expression was visible at 3 d.p.l. (*f*). (*g*) Quantification of the number of ATF3 positive FMNs per section at different times after facial nerve injury. (*h*) Analysis of *Atf3, Atf2, Atf4* and *Atf6* mRNA abundance in FNs without and 3 d.p.l. in wt and *Atf3* mutant mice. Only *Atf3*, but no other family member, was induced by facial nerve injury in wt neurons. No compensatory expression of *Atf2, Atf4* or *Atf6* was observed in *Atf3* mutant animals. (*i,j*) Unlesioned (*i*) and lesioned (*j*) facial nerves were stained for ATF3 expression. Without injury (*i*), no ATF3 expression was observed. By contrast, ATF3 was present in Schwann cells of an injured facial nerve (arrows in *j*). *n* ≥ 3 animals each bar. Data are presented as mean ± s.d. ***p ≤ 0.001. Scale bar (*a–f*) = 100 µm; (*i,j*) = 50 µm.

Constitutive *Atf3* mutant mice (*Atf3^−^*^/−^) on a C57BL/6 background were a kind gift of Dr T. Hai (Ohio State University, USA). The *Atf3* mutant allele lacks exon B, which contains the AUG initiation codon and does not produce any ATF3 protein in the liver [43] or injured FN (figure 1). Genotyping followed a published protocol [43]. As control*,* offspring harbouring two wild-type (wt) *Atf3* alleles (*Atf3^+^*^/*+*^) were used. Wt and mutant animals were derived from breedings of two *Atf3* heterozygous parents (*Atf3^+^*^/*−*^). In heterozygous *Atf3* mice, ATF3 induction after injury was undistinguishable from *Atf3^+^*^/*+*^ mice, suggesting no dose-dependent effect of reducing one allele (data not shown).

### 2.2. Facial nerve transection

**Figure 2.**
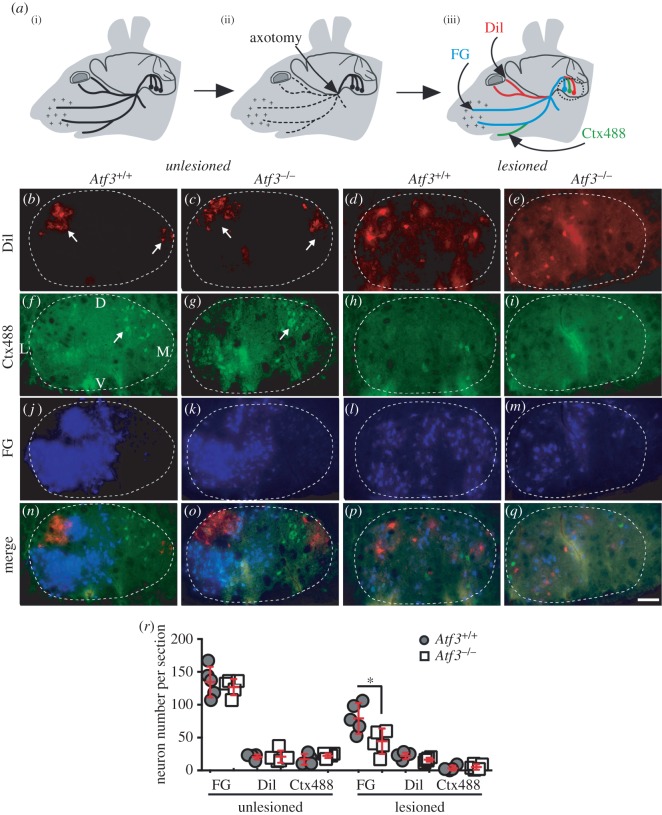
Impaired axonal regeneration in *Atf3* mutant mice. (*a*(i–iii)) The unlesioned facial nerve is depicted by a solid black line (*a*). Unilateral axotomy of the facial nerve was performed at the position marked by an arrow (*a*(ii)). Axon regeneration is quantified by injection of the fluorescent tracers DiI, FG and Ctx488 in the eyelid, whiskers and lower jaw, respectively (*a*(iii)). Upon muscle re-innervation by regenerating axons, the tracer is retrogradely transported to FMN cell bodies of the FN (black dotted circle). (*b–e*) In the unlesioned FN of wt (*b*) or *Atf3*^−/−^ (*c*) mice, FMNs connected to the eyelid were localized in two domains (arrows in *b,c*). After 23 d.p.l., DiI back-labelled FMNs were dispersed over the FN in both wt (*d*) and *Atf3^−/−^* (*e*) mice. The number of DiI positive neurons was comparable between the unlesioned and lesioned FN, suggesting rapid re-innervation of the eyelid regardless of genotype (see *r*). (*f–i*) In the unlesioned FN of wt (*f*) or *Atf3*^−/−^ (*g*) mice, retrogradely labelled Ctx488 positive FMNs were localized in a medio-dorsal FN quarter (arrows in *f,g*). Upon lesion, the number of Ctx488 positive FMNs was reduced, although comparably between wt (*h*) and ATF3-deficient (*i*) mice. (*j–m*) FG positive FMNs in unlesioned FN of wt (*j*) or *Atf3*^−/−^ (*k*) mice were restricted to the lateral half of the FN. Upon lesion, FG positive FMNs were spread all-over the entire FN in wt (*l*) and mutant (*m*) animals. However, the number of FG positive neurons was reduced in *Atf3^−/−^* (*m*) compared with wt (*l*) animals (see *r*). (*n–q*) Merged images of individual channels presented in (*b–m*). (*r*) The average number of DiI, FG or Ctx488 positive FMNs per section in unlesioned or lesioned FN was quantified at 23 d.p.l. in wt (grey circles) and ATF3-deficient (white squares) mice. FG positive neurons were significantly reduced in lesioned FN of mutant compared with wt animals. Each circle or square in (*r*) represents one mouse. The dotted lines represent the margins of the FN. L, lateral; M, medial; D, dorsal; V, ventral. Data are presented as mean ± s.d. *p ≤ 0.05. Scale bar (*b–q*) = 100 µm.

**Figure 3.**
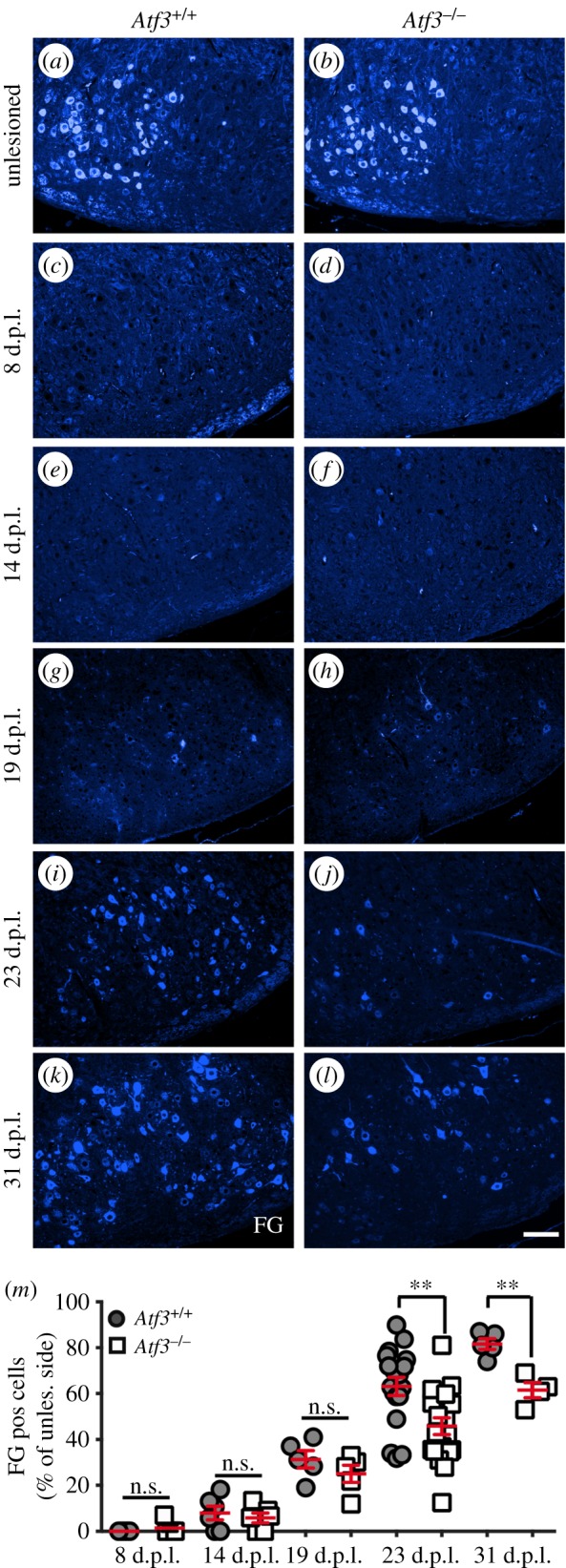
Time course of axonal regeneration in wt and *Atf3* mutant mice. (*a,b*) FG positive FMNs in the unlesioned FN of wt (*a*) and *Atf3^−/−^* (*b*) mice are restricted to the lateral half of the FN. (*c–l*) Abundance of FG labelled motoneurons in the FN of wt and *Atf3^−/−^* mice is depicted at several timepoints after lesion including 8 (*c,d*), 14 (*e,f*), 19 (*g,h*), 23 (*i,j*) and 31 (*k,l*) days post-lesion (d.p.l.). (*m*) Regeneration of FG positive FMNs was analysed at five different timepoints after injury (8, 14, 19, 23 and 31 days). The extent of regeneration is depicted as a percentage by calculating the ratio between FG positive cells counted on the unlesioned and lesioned FN. At 23 and 31 d.p.l., the percentage of FG positive neurons on the lesioned side was significantly reduced in *Atf3^−/−^* animals compared with wt. Each circle or square in (*m*) represents one mouse. Data are presented as mean ± s.d. **p ≤ 0.01. Scale bar (*a–l*) = 100 µm.

Facial nerve transection was performed as described previously [13,44]. Adult mice of either sex (approx. eight weeks old) were anaesthetized, a skin incision was made behind the right ear, and the facial nerve was exposed. Afterwards, the nerve was transected with microscissors 2 mm posterior to the *foramen stylomastoideum.* The absence of eyelid closure and whisker movement proved successful nerve transection. Regeneration of the facial nerve was quantified by retrograde axonal tracing with fluorogold (FG; Fluorochrome), 1,1′-dioctadecyl-3,3,3′,3′-tetramethylindocarbocyanine perchlorate (DiI; Molecular Probes) or choleratoxin subunit B (Ctx) conjugated with Alexa 488 (choleratoxin Alexa 488 (Ctx488); Molecular Probes). For this, 4 × l µl of FG (4% in H_2_O), 2 × 1 µl of DiI (2 µg µl^−1^ in DMSO) or 2 × 1 µl of Ctx488 (1 µg µl^−1^ in PBS) were injected with a Hamilton syringe at multiple positions in each whisker pad, eyelid or lower jaw, respectively. This injection was performed at 4, 10, 15, 19 or 27 days post-lesion (d.p.l.; figures 2 and 3). The tracers were given for 4 days for retrograde axonal transport, resulting in total regeneration periods of 8, 14, 19, 23 and 31 d.p.l., as indicated in figures 2 and 3. After those 4 days, mice were sacrificed and brains were dissected. FG, DiI or Ctx488 positive neurons of all sections of both FNs per each animal were evaluated before immunohistological staining. All experiments are in accordance with institutional regulations by the local animal ethical committee (Regierungspräsidium Tübingen, Germany).

### 2.3. Cell biology

Mouse DRG neurons were derived from adult (seven to nine weeks old) wt or *Atf3* mutant mice. For neurite growth experiments, 5 × 10^3^ neurons were plated on poly-l-lysine (PLL; 100 µg ml^−1^) and laminin (5 µg ml^−1^)-coated 12 mm coverslips. For qPCR experiments, 2.5 × 10^4^ cells were plated on PLL (10 µg ml^−1^) and laminin (2 µg ml^−1^)-coated 12-well plates. For chromatin immunoprecipitation (ChIP) experiments, 5 × 10^6^ primary P3–P5 postnatal cerebellar neurons were plated on PLL/laminin-coated 10 cm dishes. The DRG culture medium consisted of neurobasal medium (Gibco) supplemented with B27, glutamine and nerve growth factor (NGF) (50 ng ml^−1^) if not indicated otherwise. Camptothecin was added at 2 µM for 24 h. Recombinant peptides for mouse VIP (Tocris; no.1911), human gastrin releasing peptide (GRP) (Tocris; no.1789), mouse galanin (Tocris; no.2696) and mouse Wnt2b (R&D systems; no.3900-WN-025) were added at 1 nM (VIP, GRP, galanin) and 1 µg ml^−1^ (Wn2tb) for 24 h to the cultures. Neurons were cultured for 24 h for neurite growth assays or 3 days for all qPCR/ChIP experiments.

Adenoviral (AV) particles expressing ATF3 (AV-ATF3) or GFP (AV-GFP) were produced in-house at Ulm University. Viral particles (2.5 × 10^5^ for AV-ATF3 and AV-GFP) were added at 2 h after plating and left on cultures throughout the entire duration of the experiment. Electroporation of DRG neurons was performed with 3 µg DNA and 100 µl electroporation solution (Mirus). The CCL2-Cherry expression vector was described previously [45].

### 2.4. Immunostaining

Cells were fixed for 15 min in 4% PFA/5% sucrose/PBS, permeabilized for 5 min in 0.1% Triton-X-100/PBS and blocked for 30 min in 2% BSA/PBS. Neurons were stained with antibodies directed against βIII tubulin (mouse, 1 : 1000; Covance) incubated overnight at 4°C. The primary antibodies were detected by Alexa488 conjugated secondary antibodies (1 : 1500; Invitrogen). F-actin was stained with Texas Red-X phalloidin (1 : 100; Biotium) added to the secondary antibody solution.

Brains, human nerves or mouse facial nerves were fixed in 4% formaldehyde (FA) for 3 days, followed by preparation of 5 µm paraffin microtome slices. For anti-CD45 and anti-CD4 stainings, 20 µm cryostat sections of unfixed brains were used. Sections were post-fixed with 4% PFA for 10 min and processed for immunofluorescence staining according to standard protocols. Immunohistochemistry was performed using biotin (1 : 500; Vector Laboratories) or Alexa Fluor (1 : 500; Invitrogen) conjugated secondary antibodies and peroxidase-based detection systems using the ABC complex (Vector Laboratories) and DAB as substrate. Primary antibodies included anti-ATF3 (rabbit, 1 : 2000, # HPA001562; Atlas Antibodies), anti-FG (rabbit, 1 : 5000, AB153; Millipore), anti-ionized calcium-binding adapter molecule 1 (IBA1) (rabbit, 1 : 1000, 019-19741; WAKO) anti-GFAP (mouse, 1 : 1000, sc-33673; Santa Cruz Biotechnology), anti-CD45 (mouse, 1 : 100; BD Pharmingen), anti-CD4 (rat, 1 : 100; BD Pharmingen), anti-galanin (rabbit, 1 : 1000, T-4334; Bachem), anti-S100 (mouse, 1 : 200; Abcam) and anti-CCL2 (rabbit, 1 : 100, 500-P113; Peprotech). Informed consent was obtained from all patients included in the study.

### 2.5. Transmission electron microscopy

**Figure 4.**
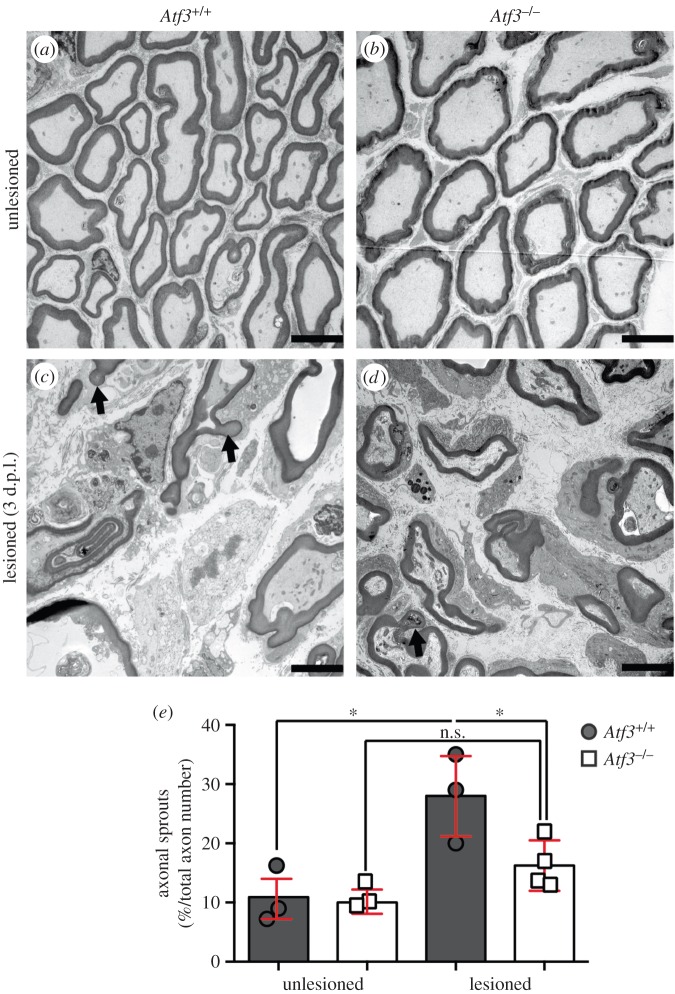
Analysis of facial nerve sprouting by transmission electron microscopy. (*a–d*) Three days after injury, unlesioned (*a,b*) and lesioned (*c,d*) facial nerves of wt (*a,c*) and *Atf3* mutant (*b,d*) animals were analysed by EM for the presence of nerve sprouts. In unlesioned nerves, only few axons contained sprout-like structures (*a,b*). By contrast, lesioned axons contained axonal sprouts (arrows in *c,d*) with a neck and bulb-like terminal structure (*c,d*). (*e*) Quantification of the percentage of axons with sprouts in relation to the total number of axons. In lesioned nerves of wt mice, the number of axons with sprouts was significantly elevated compared with nerves derived from ATF3-deficient animals. Each circle or square represents one mouse. Data are presented as mean ± s.d. *p ≤ 0.05. Scale bar (*a–l*) = 5 µm.

Facial nerve parts adjacent to the transection position were harvested 3 days after lesion. In addition, unlesioned control nerves were collected of the same animals. Samples were fixed with 2.5% glutaraldehyde in 0.1 M phosphate buffer (pH 7.2) with 1% saccharose overnight and washed with PBS and contrasted with 2% aqueous osmium tetroxide for 1 h. Increasing 1-propanol concentration series (30, 50, 70 and 90%) were used to dehydrate samples, contrasted with saturated alcoholic uranyl acetate solution for 30 min at 37°C and embedded in Epon resin. The sections were cut using the Ultracut UCT ultramicrotome (Leica) using a diamond knife (Diatome, Biel, Switzerland) and mounted on copper grids for transmission electron microscopy (TEM). Samples were analysed at an acceleration voltage of 80 kV in a transmission electron microscope (Zeiss EM 10) after contrasting the sections with lead citrate for 1 min. We analysed a total of 365, 354, 145 and 230 axons in wt unlesioned (*n* = 3), *Atf3* mutant unlesioned (*n* = 3), wt lesioned (*n* = 3) and *Atf3* mutant lesioned (*n* = 4) animals, respectively. Sprouts were identified by a bulb-shaped tip morphology with a neck structure clearly separated from the parental axon according to sprout structures described in a previous EM study [46]. To distinguish axonal sprouts from axonal protuberances, axonal sprouts had to show a clear budding off morphology from the parental axon (examples are labelled with arrows in figure 4). The number of axonal sprouts was normalized to the total number of axons present in each frame. For each nerve, at least eight different pictures at 3000× magnification were analysed.

### 2.6. Transcriptomics, laser microdissection and quantitative real-time PCR (qPCR)

FN of individual animals were dissected from 300 µm brainstem sections prepared with a tissue chopper using tungsten needles. Total RNA was isolated with the mini RNeasy kit (Qiagen) or peqGOLD MicroSpin Total RNA Kit (Peqlab). For transcriptomics, we subjected a total of four samples (wt lesion, wt no lesion, *Atf3* mutant lesion and *Atf3* mutant no lesion) to microarray analysis. In each sample, four animals were pooled to obtain sufficient amounts of mRNA (16 animals in total). Thus, although four animals were pooled for one sample, the number of biological replicates is one in figure 6 and no statistical testing was possible. However, four independent biological replicates per condition were used to confirm transcriptomics results in the subsequent qPCR analysis (figure 7). Here, statistical testing was included.

For microarray analysis, 200 ng total RNA was used as starting material and 5.5 µg ssDNA per hybridization (GeneChip Fluidics Station 450; Affymetrix, Santa Clara, CA, USA). The total RNAs were amplified and labelled following the Whole Transcript (WT) Sense Target Labeling Assay (http://www.affymetrix.com). Labelled ssDNA was hybridized to Mouse Gene 1.0 ST Affymetrix GeneChip arrays (Affymetrix). The chips were scanned with an Affymetrix GeneChip Scanner 3000 and subsequent images analysed using Affymetrix^®^ Expression Console™ Software (Affymetrix). Raw feature data were normalized and intensity expression summary values for each probe set were calculated using robust multiarray average [47].

For laser microdissection, brains were dissected and frozen without fixation. Brain cryosections (20 µm) containing the FN were prepared on membrane slides (Zeiss MembraneSlide-1.0 PEN Cat.#: 415190-9041-000), fixed for 1 min with 70% EtOH and stained with a 1% cresyl-violet solution in 50% ethanol/DEPC treated water. Motoneurons or neighbouring tissue of the FN were collected using the PALM Microbeam Laser capture equipment on a Zeiss Axiovert 200 M microscope frame. Cells from approximately 15 sections per mouse were laser microdissected. RNA was prepared using the RNeasy Plus Micro kit (Qiagen; no.74034). All materials and solutions used were treated to be RNAse free.

For cDNA synthesis, reverse transcription was performed with approximately 150–400 ng of RNA using reverse transcriptase (Promega) and random hexamers. qPCR was performed on a Roche LightCycler^®^ 480 with the SYBR Premix ExTaq (Tli RNase H Plus) PCR Master Mix (TaKaRa). Expression was determined in relation to *Gapdh* RNA levels. Primer sequences are provided in the electronic supplementary material.

### 2.7. Chromatin immunoprecipitation

We followed a previously published protocol [48]. Primary P3–P5 postnatal cerebellar cultures were fixed for 15 min at room temperature with 1% FA in PBS. We used 2 µg ml^−1^ anti-ATF3 (Santa Cruz; SC-335, rabbit) or IgG antibody for each IP. After purification of DNA (PCR purification kit, Qiagen), 2 µl of each input or IP were subjected to qPCR with primers provided in the supplement. Ct values obtained with ATF3 or IgG ChIP were normalized to the respective input values.

### 2.8. Statistical analysis

Numbers (*n*) of cell cultures or animals are indicated in figure bars or text. For cell culture, at least three independent experiments were performed and at least 30 neurons were analysed in each experiment. Neurite growth was quantified with the NeuriteTracer plugin of ImageJ [49]. For quantification of immune cells, we used the Keyence BZ Analyzer software with the Hybrid cell count tool. For this, an ellipse of 950 × 750 µm was placed over the FN area. Objects below 5 µm^2^ were discarded. For IBA-1/CD45 positive or GFAP positive cells, a threshold of 50 or 80, respectively, was used. The software calculated the total area (in square micrometres) of all objects positive for a given marker above these thresholds.

Statistical significance was calculated using Prism6 software with two-way ANOVA multiple comparison tests, with *, **, *** indicating *p* ≤ 0.05, 0.01 and 0.001, respectively; s.d. is provided if not mentioned otherwise.

## 3. Results

### 3.1. Temporal and spatial profile of ATF3 induction upon facial nerve axotomy

So far, ATF3 upregulation after injury was demonstrated at single timepoints after facial nerve lesion [37–42]. We analysed ATF3 expression in FMNs at several timepoints after unilateral axotomy in wild-type (wt) mice (figure 1). In the absence of a lesion (figure 1*a*) as well as at 6 h post-lesion (h.p.l.; figure 1*b*), no ATF3 signal was detectable in FMNs (quantified in figure 1*g*). The maximal number of ATF3 positive FMNs was observed at 3 d.p.l. (figure 1*c*) and was already slightly weaker at 7 d.p.l. (figure 1*d*). After 21 d.p.l., ATF3 was no longer present in FMNs (figure 1*e,g*).

Besides wt mice, we also analysed *Atf3* mutant mice (figure 1*f,h*). At 3 d.p.l., ATF3 protein levels were absent in *Atf3* mutant mice (figure 1*f*). In agreement, qPCR analysis of mRNA levels revealed no *Atf3* induction in *Atf3* mutant FN at 3 d.p.l. (figure 1*h*). By contrast, *Atf3* mRNA was strongly upregulated upon lesion in wt mice (figure 1*h*), similar to ATF3 protein (figure 1*c*). In this injury model, other ATF/CREB family members (*Atf2, Atf4, Atf6)* were not modulated by nerve injury in wt mice (figure 1*h*), as opposed to other injury models [50]. Also, the ATF/CREB family member *Creb* was not altered by ATF3 deficiency (data not shown). Further, no compensatory upregulation of these ATF/CREB members was observed in *Atf3* mutant mice (figure 1*h*).

Besides FMN intrinsic responses, repair programmes are also initiated in Schwann cells [51,52]. Because ATF3 is upregulated upon injury in Schwann cells of the sciatic nerve [53,54], we wondered whether ATF3 expression is also induced in facial nerve Schwann cells. Indeed, we observed an increase in ATF3 in facial nerve Schwann cells in wt mice at 3 d.p.l. (see arrows in figure 1*j*) compared with the unlesioned facial nerve (figure 1*i*). However, the number of ATF3 positive cells and signal intensity of ATF3 in the lesioned facial nerve were weaker compared with injured FMNs (figure 1*g*).

Overall, we observed a strong ATF3 upregulation in FMNs and to a weaker extent also in Schwann cells occurring in the first week after PNS injury.

### 3.2. Axonal regeneration is reduced in *Atf3* mutant mice

We analysed the extent of axonal regeneration in adult wt and *Atf3* mutant mice at several timepoints after unilateral facial nerve transection (figures 2 and 3). Responses in the spared contralateral FN served as intra-animal control. Similar to the retino-tectal system [55], FMNs are topographically organized. Here, different FMN populations, e.g. those connected to the eyelid, whisker pad and lower jaw, are confined to specific FN subdomains [56–58].

We analysed the regeneration potential of these different FMN populations and the role of ATF3 in this regeneration process with FMN back-labelling experiments using three different fluorescent tracers (figure 2*a*(i–iii)). FMNs innervating the eyelid, whiskers and lower jaw were retrogradely labelled through DiI (red), FG (blue) and choleratoxin Alexa 488 (Ctx488, green) injection, respectively (figure 2*a*(iii)). Injections were performed on the unlesioned and lesioned sides to determine the FMN topographic map before and after lesion. Impaired tracer transport along transected facial nerves to FMNs indicates loss of axonal re-growth to their original postsynaptic muscle targets [13,16]. Of note, this retrograde tracing method allows following of the regeneration process only after facial muscles have been re-innervated by regenerating axons. By contrast, first axon outgrowth responses before reaching muscle targets are not detected by this method.

On the unlesioned side, FMNs were topographically localized as reported before [56–58]. DiI positive FMNs connected with the eyelid occupy two domains (arrows figure 2*b,c*), whereas FMNs connected to the lower jaw were consistently localized to the dorsomedial FN quarter (arrows in figure 2*f,g*). Finally, FG positive FMNs innervating the whisker pad were restricted to the lateral half of the FN (figure 2*j,k*). This FG positive FMN subtype representing the whiskers has the highest numbers in the FN compared with DiI and Ctx488 positive FMNs (figure 2*n* or *o*; quantified in *r*). Thus, eyelid and lower jaw are represented by fewer FMNs, in line with previous reports [56–58]. Comparing FMN localization and numbers between wt (figure 2*n*) and ATF3-deficient (figure 2*o*) mice, no obvious differences were discernible on the unlesioned side (quantified in figure 2*r*).

Next, we inspected the lesioned FN. After 23 d.p.l., a disorganization of the FN topographic map was observed for all three tracers (figure 2*p,q*). Regenerating FMN axons initially also sprout into topographically wrong target areas [56–58]. Here, they pick up different tracers, resulting in back-labelling of FMNs in topographically aberrant positions. At much later timepoints (not covered in this study), such aberrantly formed sprouts or synapses are eliminated [56–58]. When comparing the localization of individual FMNs within this disorganized topographic map, no obvious differences were observed after lesion between wt (figure 2*d,h,l,p*) and *Atf3* mutant (figure 2*e,i,m,q*) animals.

Subsequently, we quantified, the number of regenerating FMNs of these different FMN subtypes by counting DiI, FG and Ctx488 positive FMNs on the lesioned side of wt and *Atf3* mutant animals (figure 2*r*). Interestingly, at 23 d.p.l., DiI positive FMNs connected to the eyelid regenerated almost to 100%, however with no difference between genotypes (figure 2*b–e*; *n–q*; quantification in *r*). In contrast with this, only approximately 30% of FMNs innervating the lower jaw regenerated in both wt and mutant animals at the same timepoint (figure 2*f–i*; quantified in *r*). Here, overall neuron numbers on the lesioned side were low (figure 2*h,i*), rendering quantification more difficult (figure 2*r*). Finally, we inspected the largest FMN population and observed that in wt mice approximately 60% of FG positive FMNs were reconnected to the whisker muscles (figure 2*j–m*,*r*). Importantly, for this FMN subtype, we observed a significant difference in axon regeneration between wt and ATF3-deficient animals. Now, only an average 35% of neurons was FG positive in *Atf3* mutant animals (figure 2*m,q,r*). This finding indicates a role of ATF3 in stimulating axon regeneration of lesioned FMNs.

In order to analyse axon regeneration in *Atf3* mutant animals more comprehensively, we provide a temporal regeneration profile including a total of five timepoints after lesion (8, 14, 19, 23 and 31 d.p.l.), focusing on FG positive FMNs (figure 3). For quantification, a percentage of regenerated neurons was determined, by calculating the ratio between FG positive FMNs on the lesioned and unlesioned FN (figure 3*m*). Inspection of wt mice revealed a correlation of the regeneration efficiency of FG positive FMNs with the timespan allowed for regeneration after injury. At 8 d.p.l., no FG positive neurons were observed (figure 3*c*), whereas 10 and 30% of FMNs were tracer positive at 14 (figure 3*e*) and 19 (figure 3*g*) d.p.l. in wt mice, respectively (figure 3*m*). Thus at injury timepoints before 14 days, axons may have started regeneration but have not yet reached their whisker muscle targets. At later timepoints, more robust re-innervation of facial muscles by regenerating axons was observed in wt mice. Now, 65 and 82% of FMNs were FG positive in the lesioned FN, at 23 (figure 3*i*) or 31 (figure 3*k*) d.p.l., respectively (figure 3*m*).

In *Atf3* mutant mice, no FG positive FMNs were observed at 8 d.p.l., similar to wt mice (figure 3*d*). At 14 d.p.l. injury, no difference in FG positive FMNs was observed between wt and ATF3 lacking mice (figure 3*f*). At 19 d.p.l., the percentage of regenerated FMNs in *Atf3* mutant mice was slightly decreased compared with wt (figure 3*h,m*). This difference was further pronounced at 23 and 31 days post lesion. At these timepoints, 45 and 60% of FMNs were regenerating at 23 and 31 d.p.l., respectively, resulting in a significant reduction of FG positive FMNs at both timepoints in *Atf3* mutant compared with wt mice (figure 3*j,l,m*).

So far, axonal regeneration responses were only analysed in the FN. Besides the FN, we investigated the presence of axonal sprouts in the facial nerve 3 days after injury (figure 4; *n* ≥ 3 nerves per condition).

In unlesioned wt (figure 4*a*) and *Atf3* mutant (figure 4*b*) facial nerves, approximately 10% of all axons had clearly distinguishable sprout-like structures consisting of a bulb and a neck separated from the parental axon (quantified in figure 4*e*). Three days after facial nerve lesion, the number of nerve sprouts was elevated in wt (arrows in figure 4*c*) and *Atf3* mutant (figure 4*d*) animals. Now, approximately 30% of all lesioned wt axons protruded sprout-like structures, whereas only 16% of nerves derived from ATF3-deficient animals showed these protrusions (figure 4*e*). This finding on axonal sprouts in the facial nerve suggests an influence of ATF3 on facial nerve regeneration at an early timepoint after nerve injury.

Taken together, a first analysis of ATF3-deficient mice showed reduced PNS axon regeneration.

### 3.3. Facial motoneuron number and immune responses are not affected by ATF3 ablation

**Figure 5.**
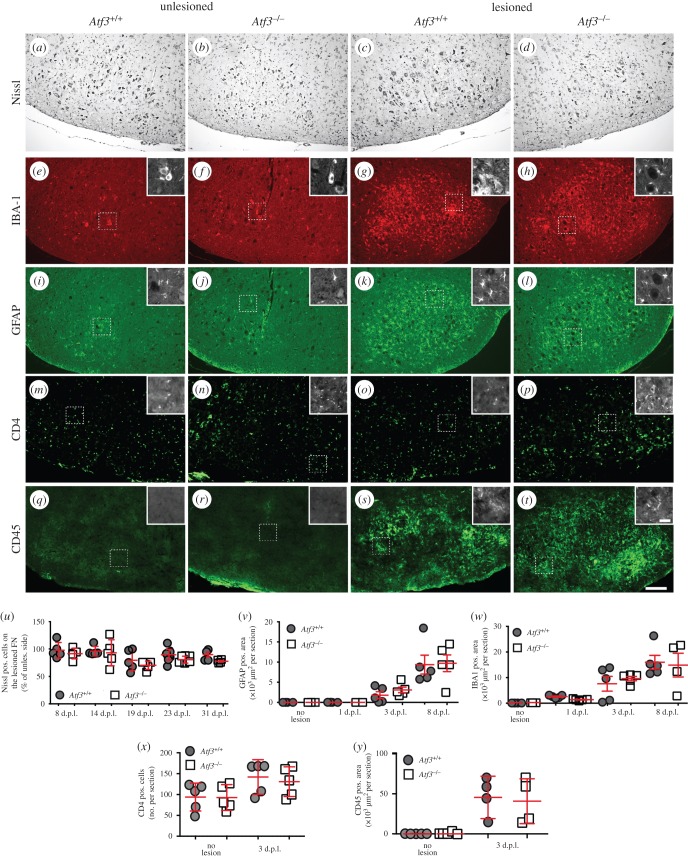
FMN numbers and immune responses are unaltered in *Atf3*^−/−^ animals. (*a–d*) Nissl staining was employed to label all FMNs present in FN. No major FMN loss was observed upon lesion in wt (*c*) or *Atf3*^−/−^ (*d*) mice. (*e–l*) Microglia (*e–h*) and astrocytes (*i–l*) were labelled at 8 d.p.l for IBA-1 and GFAP expression, respectively. In the uninjured FN of wt (*e,i*) or ATF3-deficient (*f,j*) animals, no IBA-1 positive microglia (*e,f*) or GFAP positive astrocyte (*i,j*) cell infiltration was observed. By contrast, upon injury (*g,h* and *k,l*) there was a comparable microglia and astrocyte activation in wt (*g,k*) and *Atf3* mutant (*h,l*) animals. (*m–p*) CD4^+^ cells were found on the unlesioned side of wt (*m*) and *Atf3* mutant (*n*) animals. CD4^+^ T cells were slightly upregulated upon lesion in wt (*o*) as well as *Atf3* mutant (*p*) FNs. (*q–t*) CD45, a marker for CNS infiltrating monocytes, was nearly absent on the unlesioned FN (*q,r*). Facial nerve lesion increased CD45 positive cells on the FN of wt (*s*) and *Atf3* mutant (*t*) animals. Insets in (*e–t*) show higher magnifications of areas marked with a dashed box. (*u–y*) The percentage of Nissl positive FMNs on the lesioned FN (control side set to 100%; *u*), total area (in square micrometres) of GFAP positive astrocytes (*v*), number of IBA1 positive microglia (*w*), number of CD4^+^ cells (*x*) or total area (in square micrometres) of all CD45^+^ cells (*y*) per section were counted and plotted against the different timepoints. Each circle or square in (*u–y*) represents one mouse. Data are presented as mean ± s.d. Scale bar (*a–t*) = 100 µm; insets (*e–t*) = 5 µm.

An obvious factor affecting the outcome of axonal regeneration would be differential FMN loss between wt and *Atf3*^−/−^ mice. Using Nissl staining to visualize all FMNs present in a FN, we did not observe major differences between genotypes at all injury timepoints analysed (figure 5*a–d*; quantified in *m*). Thus, differential FMN loss is not obviously responsible for alterations in axonal regeneration observed in ATF3-deficient mice (figures 2 and 3).

We also investigated whether ATF3 ablation interfered with induction of a PNS lesion-associated immune response (figure 5*e–l*,*v*,*w*). Upon facial nerve injury, GFAP positive astrocytes and IBA-1 positive microglia are reported to infiltrate the lesioned but not the intact FN [40]. Accordingly, almost no astrocytes or microglia cells were observed in the unlesioned FN of wt (figure 5*e,i*) or ATF3-deficient (figure 5*f,j*) mice. By contrast, both brain-resident immune cells were present in the lesioned FN at 8 d.p.l. Astrocyte and microglia numbers were indistinguishable between wt (figure 5*g,k*) and *Atf3* mutant (figure 5*h,l*) mice (figure 5*v,w*). A similar finding was observed at other injury timepoints (figure 5*v,w*).

Besides brain-resident immune cells we inspected CD4 and CD45 positive cells, labelling T-cells and CNS infiltrating monocytes, respectively (figure 5*m–t,x,y*). In unlesioned FN, CD4^+^ cells were found in wt (figure 5*m*) and *Atf3* mutant (figure 5*n*) mice. After three days of lesion, CD4^+^ cells were slightly but not significantly upregulated on the lesioned side. However, no difference was observed between genotypes (figure 5*o,p*; quantified in *x*). CD45 positive cells were nearly absent in the unlesioned FN (figure 5*q,r*). Upon lesion, CD45 positive cells were upregulated on the lesioned side (figure 5*s,t*), however in a comparable manner between wt and *Atf3* mutant mice (figure 5*y*).

This suggests no obvious impact of ATF3 deletion on the injury-mediated brain immune response.

### 3.4. Genome-wide identification of ATF3-dependent genes upon facial nerve injury

**Figure 6.**
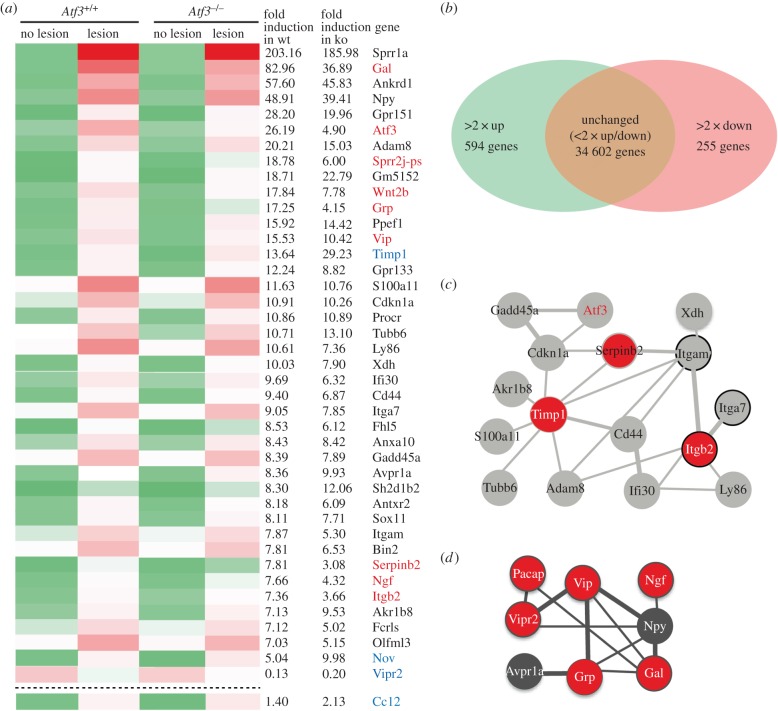
Transcriptomics analysis of wt and ATF3-deficient facial motor nuclei. (*a*) Heatmap of genes most strongly (more than sevenfold) altered by facial nerve injury in wt or ATF3-deficient mice. The fold induction in wt and *Atf3* mutant mice was calculated in relation to expression values on the uninjured site. Genes depicted in black were not obviously affected by ATF3 deficiency. Expression of genes labelled in red or blue colour was reduced or elevated in *Atf3* mutant compared to wt mice, respectively. Green colours in bars depict weakest, whereas red colours indicate strong expression levels. (*b*) In wt mice, 849 genes out of over 35 000 genes present on the microarray were regulated more than twofold. The number of genes upregulated by injury (594) exceeded those downregulated (255) more than twofold. (*c,d*) STRING networks of protein clusters identified in wt mice upon facial nerve injury. A first protein cluster (*c*) revealed known ATF3 interaction partners such as Cdkn1a and Gadd45a and further interactions of ATF3 with for instance components of integrin signalling (*Itgam, Itgb2 Itga7*). A second protein network (*d*) consisted of neuropeptide signalling components such as neuropeptides (*Pacap, Vip, Ngf, Npy, Grp, Gal*) and receptors (*Avpr1a, Vipr2*). Genes labelled in red were affected by ATF3 deficiency.

The *Atf3* gene encodes a RAG-associated TF that may induce expression of further effector RAGs upon injury. So far, no genome-wide data are available on the impact of ATF3 loss-of-function on gene expression. In order to analyse the impact of ATF3 deletion on gene expression, we employed transcriptomics with unlesioned and lesioned FNs of wt and *Atf3* mutant mice 3 d.p.l. (figure 6).

In the lesioned FN of wt mice, we observed an induction of a RAG encoding gene regulatory network encompassing several hundreds of genes. The heatmap in figure 6*a* depicts those genes being up or downregulated in injured wt mice by at least a factor of seven. Notably, the RAG cluster induced by injury in wt mice in this study was almost identical to a previous independent transcriptomics study [40], thereby underscoring specificity and reproducibility of results presented in this study. Prototypical RAGs most strongly induced by nerve injury were *Sprr1a, Galanin, Npy, Vip, Atf3*, *Sprr2j, Cdkn1a* (p21) and *Itga7* (depicted in figure 6*a*). In addition, other genes including *Gpr151, Wnt2b, Grp,* TIMP metallopeptidase Inhibitor (*Timp*)*, Gpr133* and *Serpinb2*, so far not considered as prototypical RAGs, were also induced by facial nerve injury in wt mice (figure 6*a*).

Out of the more than 35 000 transcripts present on the microarray, the majority of genes (34 602) was largely unchanged or modulated only below a twofold change (less than or equal to twofold) between control and lesioned FN (figure 6*b*). Among the genes regulated more than twofold, 594 genes were induced, whereas only 255 genes were downregulated upon lesion in wt mice 3 days after injury (figure 6*b*). The ratio between gene up and downregulation was even more pronounced when focusing on genes with at least sevenfold changed mRNA abundance in wt mice (depicted in figure 6*a*). Here, 40 genes were upregulated, in contrast with a single gene (*Vipr2*) being downregulated. This suggests that nerve injury favours gene induction over gene repression.

Employing STRING (Search Tool for the Retrieval of Interacting Genes/Proteins) analysis of the top regulated genes, ATF3 was directly or indirectly connected with other genes induced by facial nerve injury in wt mice (figure 6*c*). These included integrin-associated signalling members such as *Itga7a, Itgb2* and *Itgam* and the cell cycle regulator p21 (*Cdkn1a*). STRING analysis and GO (Gene Ontology) annotation uncovered a further module of functionally related genes modulated by facial nerve injury (figure 6*d*; electronic supplementary material, table S1). This cluster contained members of neuropeptide signalling pathways such as *Vip, Ngf, Galanin, Npy, Grp, Pacap* (*Adcyap1*) and *Avpr1a* all of which, except for the VIP receptor *Vipr2,* being strongly induced by facial nerve injury in wt mice (figure 6*a*,*d*).

Inspection of *Atf3* mutant mice revealed that many genes modulated by facial nerve injury in wt mice were in general also induced upon ATF3 deletion' however not to the same extent. For instance, approximately 25% of the strongest regulated genes (at least sevenfold) depicted in figure 6*a* were at least 1.5-fold altered between lesioned wt and *Atf3* mutant animals. In addition, out of the 594 and 255 genes being twofold up or downregulated in wt mice (figure 6*b*), 135 and 55, respectively were altered at least twofold in *Atf3* mutant mice (electronic supplementary material, table S1). Hence, approximately 20–25% of all genes regulated by facial nerve injury in wt mice were to a certain extent ATF3 dependent at 3 days post-injury.

Besides this general impact of ATF3 on the RAG response, specific RAGs were strongly affected by ATF3 deficiency. For instance, *Galanin*, *Sprr2j* but also *Wnt2b, Grp, Vip, Serpinb2, Itgb2* and *Ngf* had reduced mRNA abundance in severed *Atf3* mutant compared to wt FN (labelled in red in figure 6*a*). As mentioned above, we noted induction of a cluster of eight neuropeptide signalling encoding genes in injured wt mice (figure 6*a,d*). Notably, expression of six of these genes (*Vipr2, Vip, Ngf, Grp, Gal, Adcyap1)* was reduced in *Atf3* mutant tissue suggesting a role of ATF3 in wt mice for induction of these RAGs (red in figure 6*d*). Besides ATF3-mediated gene induction, other genes such as *Timp1,* nephroblastoma overexpressed gene (*Nov*) (also called *Ccn3*) and *Ccl2* had elevated mRNA levels in *Atf3* mutant mice compared with wt (labelled in blue in figure 6*a*). This indicates ATF3-dependent gene repression of these genes in wt mice.

**Figure 7.**
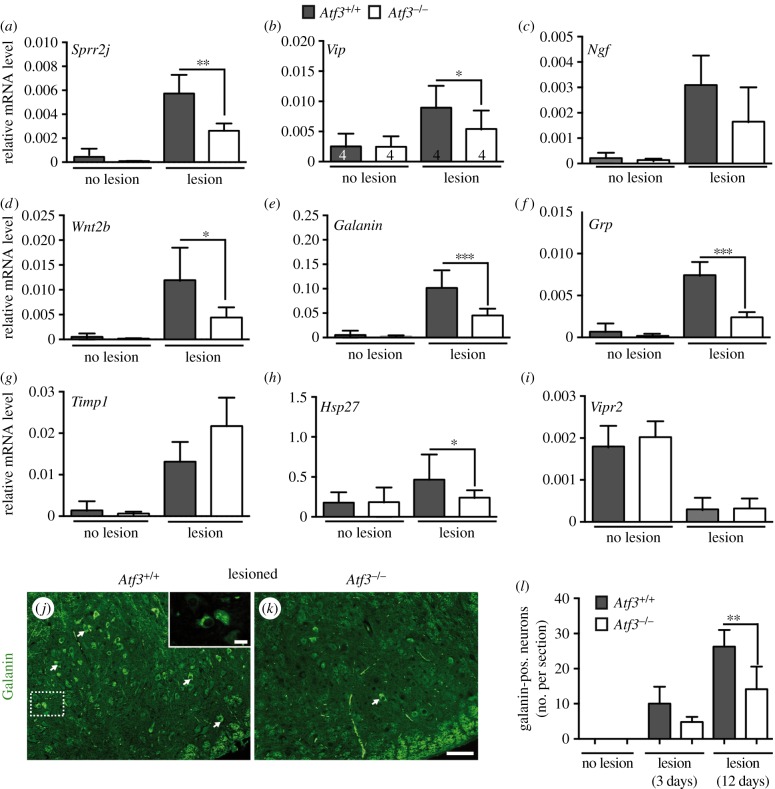
ATF3 regulates injury-related expression of neuropeptides and other genes. (*a–i*) Unlesioned and injured FN of *Atf3*^+/+^ and *Atf3*^−/−^ mice were subjected to qPCR analysis after 3 days of injury to quantify mRNA abundance of primers indicated. In wt mice (grey bars), facial nerve injury resulted in induction of *Sprr2j* (*a*), *Vip* (*b*), *Ngf* (*c*), *Wnt2b* (*d*), *Galanin* (*e*), *Grp* (*f*), *Timp1* (*g*) and the known ATF3 target gene *Hsp27* (*h*). In contrast to wt mice, induction of *Sprr2j, Vip, Ngf, Wnt2b, Galanin, Grp* and *Hsp27* mRNA abundance was reduced upon facial nerve lesion in *Atf3* mutant mice (white bars). *Timp1* mRNA induction was more pronounced upon ATF3 loss-of-function (*g*). The Vip2 receptor (*Vipr2*) was downregulated by facial nerve injury in wt and ATF3-deficient mice (*i*). Numbers in bars in (*b*) reflect independent biological replicates for experiments in (*a–i*). (*j–l*) Confirmation of reduced galanin expression in ATF3-deficient mice upon facial nerve injury. Deafferented wt (*j*) and ATF3-deficient (*k*) FN were stained with anti-galanin directed antibodies. In wt mice (*j*), galanin localized in secretory vesicle-like structures (see inset) of FMNs (some labelled with an arrow). The number of galanin immunoreactive FMNs was reduced in *Atf3* mutant mice (*k*). (*l*) Quantification of galanin positive neurons without and 3 and 12 days after lesion. Data are presented as mean ± s.d. **p ≤ 0.01. Scale bar (*j,k*) = 50 µm; inset = 5 µm.

In a next step, transcriptomics data were corroborated by qPCR with independent cDNAs derived from different wt and *Atf3* mutant animals (figure 7). In agreement with microarray results (figure 6*a*), mRNA abundance of *Sprr2j, Vip, Ngf, Wnt2b*, *Galanin* and *Grp* was strongly augmented in deafferented FN of wt mice but significantly reduced in lesioned *Atf3* mutant FN (figure 7*a–f*). We also confirmed microarray data for *Vipr2* and *Timp1* (figure 7*i,g*).

In principal, we would expect that the identified RAG response occurs predominantly in injured FMNs. However, besides FMNs, other cell types, e.g. immune cells (figure 5), were also present in the lesioned FN and might contribute to the transcriptional changes observed. In order to test, whether FMNs were the main cell type responsible for transcriptional changes after facial nerve injury, we performed laser microdissection experiments (electronic supplementary material, figure S1). Here, FMNs of the unlesioned and lesioned FN were dissected 3 d.p.l. In addition, we included neighbouring tissue on the unlesioned and lesioned sides not containing FMNs (electronic supplementary material, figure S1). Indeed, inspection of eight genes strongly induced by facial nerve transection in our transcriptomics analysis (figure 6) revealed strong mRNA upregulation only in the sample containing FMNs on the lesioned side but not in all other three samples (electronic supplementary material, figure S1). This suggests that the majority of transcriptional changes observed in the microarray analysis (figure 6) took place in FMNs and not surrounding cells.

So far, we focused on transcriptional changes after 3 days post-injury (figure 6; electronic supplementary material, figure S1). In order to analyse the time window of injury-regulated gene induction, we inspected lesioned FNs at 7 and 14 days post-injury by qPCR focusing on eight injury-regulated genes (electronic supplementary material, figure S2). After 7 days of injury, mRNA levels of the majority of genes analysed (seven out of eight) were still above control levels and reached significance for *Galanin, Ngf, Grp, Sprr1a* and *Sprr2j* (electronic supplementary material, figure S2). One week later, at 14 d.p.l., mRNA abundance was clearly decreased and only selected genes such as *Galanin, Grp* and *Sprr1a* were elevated (electronic supplementary material, figure S2). Thus, a facial nerve injury-induced RAG response appears to last for at least 7 days after lesion, with mRNA abundance of selected RAGs persisting up to 14 days post-injury.

In addition, we inspected the reported ATF3 target gene *Hsp27* [12,59]. In line with previous reports demonstrating *Hsp27* induction by ATF3 overexpression [12], we observed reduced *Hsp27* abundance in ATF3-deficient animals upon injury (figure 7*h*). We also analysed other prototypical RAGs encoding for transcription factors (*c-Jun, Smad1, Stat3*, *Sox11*) or effector RAGs (*Cap23, Scg10, Gap43, Npy*). All these RAGs were induced upon facial nerve lesion, however indistinguishably between wt and *Atf3* mutant mice except for *Cap23* and *Nov* (electronic supplementary material, figure S3 and table S1).

In order to confirm mRNA changes observed for the gene encoding the galanin neuropetide at the protein level, lesioned FNs were stained with galanin-directed antibodies at 3 d.p.l. (figure 7*j,k*). In wt mice, many deafferented FMNs were positive for galanin (arrows figure 7*j*). Higher magnification suggested the presence of galanin in secretory vesicle-like structures (inset figure 7*j*). In contrast to this, the number of galanin positive neurons was reduced in ATF3-deficient FN (figure 7*k*) at two different timepoints (figure 7*l*).

Overall, this first genome-wide survey in *Atf3* mutant mice uncovered regulation of several neuropeptide-encoding genes by ATF3 as well as other potentially novel ATF3 target genes including *Wnt2b*.

### 3.5. ATF3 overexpression in peripheral nervous system neurons induces gene expression

**Figure 8.**
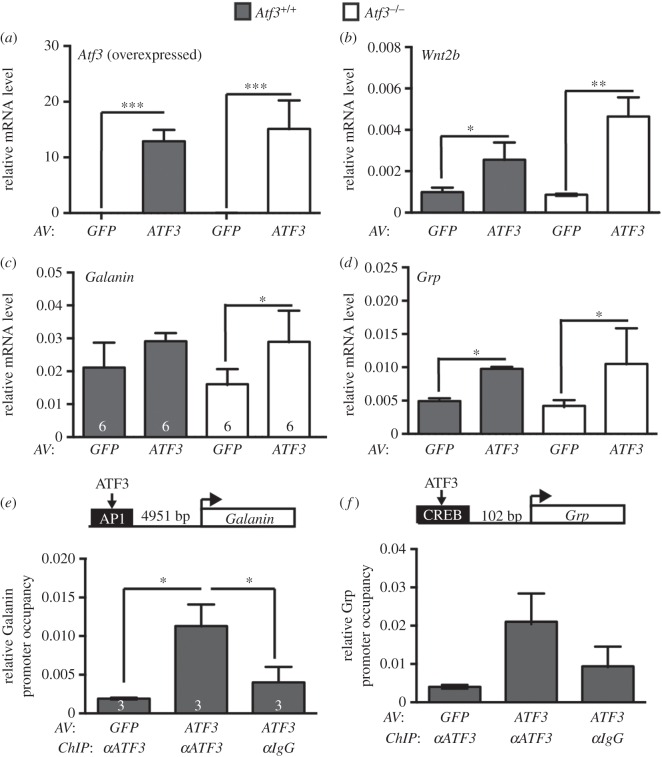
ATF3 mediates neuropeptide and *Wnt2b* expression in primary PNS neurons. (*a–d*) Adult wt or ATF3-deficient mouse DRG neurons were infected with adenoviral (AV) particles resulting in GFP (control) or ATF3 expression. mRNA levels of *Atf3* (*a*), *Wnt2b* (*b*), *Galanin* (*c*) and *Grp* (*d*) were analysed by qPCR. Viral infection strongly enhanced *Atf3* mRNA abundance in wt and *Atf3* mutant neurons (*a*). *Wnt2b* (*b*), *Galanin* (*c*) and *Grp* (*d*) mRNA levels were augmented upon viral ATF3 overexpression in wt and *Atf3* mutant DRG neurons. (*e,f*) Primary wt neurons overexpressing GFP or ATF3 were subjected to ChIP analysis with anti-ATF3 or IgG (control) antibodies. ATF3 occupancy at potential ATF3 binding sites of the *Galanin* (*e*) and *Grp* (*f*) promoter was tested with qPCR. ATF3 promoter occupancy was observed in ATF3-overexpressing samples only in the presence of anti-ATF3 but not IgG antibodies suggesting ATF3 binding at the *Galanin* (*e*) and *Grp* (*f*) promoter. Numbers in bars reflect independent numbers of experiments. Data are presented as mean ± s.d. *p ≤ 0.05; **p ≤ 0.01; ***p ≤ 0.001.

Transcriptomics data indicated a stimulatory ATF3 function in transcriptional regulation of neuropeptide expression during PNS axon regeneration (figures 6 and 7). In further experiments, we analysed whether ATF3's function in axonal regeneration might be accomplished through regulation of these neuropeptides, particularly focusing on *galanin, Grp, Vip* and *Ngf*. In addition, we included *Wnt2b*, because several Wnt family members mediate axon growth and regeneration [60,61]. To test whether genes affected by ATF3 loss-of-function were direct target genes, ATF3 overexpression and ATF3-directed ChIP were employed in wt and ATF3-deficient primary neurons (figure 8).

In the first set of experiments, ATF3 was overexpressed in adult mouse DRG neurons through adenovirus (AV) mediated infection (figure 8*a–d*). We used adult mouse DRG neurons because adult FMNs did not grow in culture (data not shown). Similar to FMNs, DRG neurons project axons to the periphery and therefore should recapitulate responses of FMNs as closely as possible. After 3 days in culture, mRNA levels of potential ATF3 target genes were quantified by qPCR (figure 8*a–d*). ATF3 was successfully overexpressed in both wt and *Atf3* mutant DRG neurons comparing AV-ATF3 with AV-GFP-infected neurons (figure 8*a*). *Sprr2j, Ngf* and *Vip* mRNA levels were not affected by ATF3 overexpression, suggesting that those genes were not under direct ATF3 control (data not shown). By contrast, *Wnt2b* (figure 8*b*), *Galanin* (figure 8*c*) and *Grp* (figure 8*d*) mRNA levels were induced by ATF3 overexpression in both wt and ATF3-depleted DRG neurons. Therefore, ATF3 overexpression upregulated *Wnt2b, Galanin* and *Grp* expression (figure 8), whereas ATF3 deficiency decreased abundance of those genes (figures 6 and 7).

Next, we analysed whether ATF3 may directly bind to promoter regions of *Wnt2b, Galanin* and *Grp*. We searched the MotifMap database [62] for ATF3 binding motifs such as an activator protein 1 (AP1), ATF3 or CREB binding motif within 5 kb up and downstream of the transcriptional start site. *Galanin* and *Grp* contained a potential AP1 and CREB binding site, respectively. *Wnt2b* contained no obvious ATF3 recognition site and was not further analysed by ChIP.

ATF3 promoter occupancy was analysed in primary neurons infected with AV-ATF3 or, as control, AV-GFP, followed by ChIP with anti-ATF3 or, as control, anti-IgG directed antibodies (figure 8*e,f*). Subsequently, qPCR was employed with primers spanning the respective potential TF binding motif. ATF3 bound to the AP1 recognition site of *Galanin* (figure 8*e*) and the CREB binding site of *Grp* (figure 8*f*) in ATF3 antibody directed ChIP samples of ATF3-expressing neurons. In contrast to this, no specific ATF3 promoter occupancy was observed in GFP-expressing neurons with the anti-ATF3 antibody or in neurons expressing ATF3 subjected to an anti-IgG directed ChIP (figure 8*e,f*). These findings suggest direct ATF3 binding to *Galanin* and *Grp* regulatory elements.

### 3.6. Impaired neurite growth in *Atf3* mutants is rescued by neuropeptide addition

**Figure 9.**
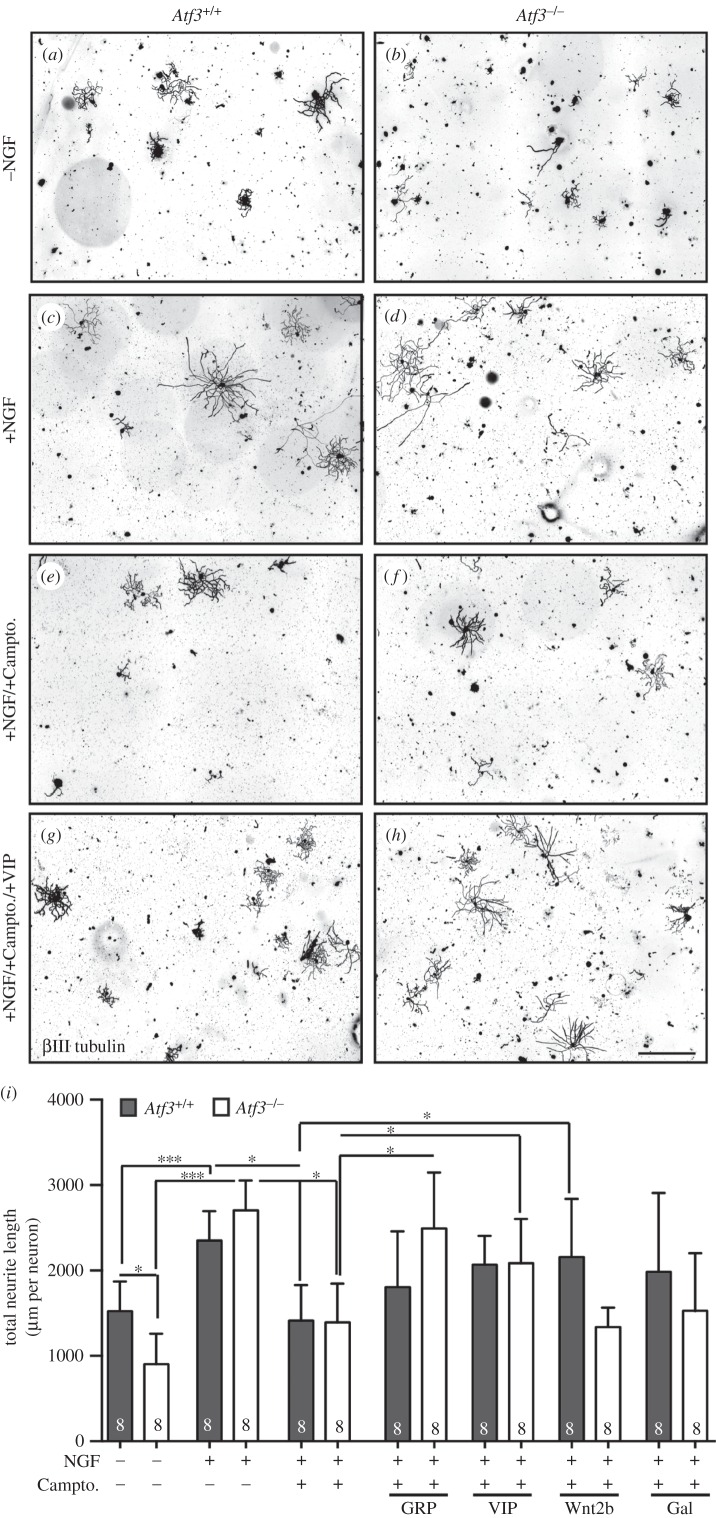
() Neuropeptide treatment elevates neurite growth upon camptothecin inhibition in ATF3-deficient mice. Adult wt or *Atf3* mutant DRG neurons were cultured for 24 h followed by labelling for neuron-specific βIII tubulin expression. (*a,b*) In the absence of NGF, wt DRG neurons (*a*) elaborated more and longer neurites compared with neurons lacking ATF3 (*b*). (*c,d*) In the presence of NGF, *Atf3* mutant (*d*) grew similarly to wt (*c*) neurons. (*e,f*) Camptothecin administration for 24 h reduced neurite growth of wt (*e*) and ATF3-deficient (*f*) neurons to a similar level. (*g,h*) Administration of the neuropeptide VIP elevated neurite growth of both wt (*g*) and ATF3-deficient (*h*) neurons in the presence of camptothecin and NGF. (*i*) Quantification of the entire neurite length/neuron for all conditions tested. In *Atf3* mutant neurons, GRP and VIP application resulted in statistically significant relief of camptothecin-mediated neurite growth inhibition. Numbers in bars reflect independent cultures analysed. Data are presented as mean ± s.d. *p ≤ 0.05; ***p ≤ 0.001. Scale bar (*a–h*) = 500 µm.

Analysis of facial nerve regeneration *in vivo* revealed decreased axon regeneration in ATF3-deficient mice (figures 2 and 3). As reduced axon regeneration might be caused by an intrinsic axon growth inhibition induced by ATF3 loss-of-function in PNS neurons, we employed *in vitro* neurite growth assays with adult wt and ATF3-lacking DRG neurons (figure 9). Besides PNS neurons, the impact of ATF3 deletion was also quantified in CNS neurons. However, in postnatal cerebellar neurons, neurite length in *Atf3* mutant compared to wt neurons was only slightly reduced (electronic supplementary material, figure S4), suggesting no strong dependence of CNS neurite growth on ATF3.

Wt DRG neurons cultured for 24 h revealed robust neurite growth (figure 9*a*). On average, the total neurite length of all neurites protruded by a single wt DRG neuron added up to approximately 1500 µm (figure 9*i*). By contrast, in ATF3-deficient DRG neurons, axon growth was reduced by approximately 40% (figure 9*b*; quantified in *i*). Here, the total length of all neurites elaborated by an individual *Atf3* mutant neuron accumulated to an average of 950 µm (figure 9*i*). These data suggest a stimulatory role of ATF3 on PNS axon growth in wt neurons and correspond with enhanced neurite growth reported upon ATF3 overexpression [8,11,12].

In the previous experiment, NGF was omitted, because *Ngf* mRNA induction was ATF3-dependent *in vivo* (figures 6 and 7) and *Ngf* mRNA levels were downregulated by approximately 50% in primary *Atf3*-deficient DRG neurons *in vitro* (data not shown). Thus, in the next set of experiments we added recombinant NGF to the culture medium and analysed whether this exogenous NGF application might rescue the neurite growth observed in *Atf3* mutant mice in the absence of NGF (figure 9*a,b*). NGF elevated neurite growth in wt neurons by approximately 30–40% (figure 9*c*) compared with neurons without NGF administration (figure 9*a*; quantified in *i*). Similar to wt neurons, NGF application also elevated axon growth in ATF3-deficient DRG neurons (figure 9*d*). In fact, neurite growth in *Atf3* mutant neurons was (approx. 10%) increased compared with wt neurons (2615 µm versus 2354 µm; figure 9*i*). This suggests that raising NGF levels in *Atf3* mutant neurons by exogenous NGF administration can rescue impaired axon growth associated with ATF3 deficiency.

In a next step, we investigated whether other ATF3-regulated neuropeptides might mediate ATF3's impact on PNS axon growth similar to NGF. Several of the ATF3-regulated neuropeptides, e.g. galanin and VIP, but so far not GRP, promote axon regeneration *in vivo* and axon growth *in vitro* [63–68]. This also holds true for several Wnt family members [69–72]; however, Wnt2b has not been analysed so far.

To test if galanin, VIP, GRP or Wnt2b are required for axon growth, recombinant galanin, VIP, GRP or Wnt2b peptides were added to wt and ATF3-lacking DRG neurons. First, we tested whether any of the neuropeptides or Wnt2b administration affected wt DRG neurite growth or rescued neurite growth in *Atf3* mutant neurons in NGF-depleted medium. However, under these conditions, none of the recombinant proteins affected wt neurite growth in a significant manner. In *Atf3* mutant neurons, only GRP administration improved slightly but not significantly neurite length (electronic supplementary material, figure S5). Thus, under physiological culture conditions, there was no overt effect of neuropeptides or Wnt2b on either wt or *Atf3* mutant neurite growth.

In order to investigate whether neuropeptides or Wnt2b modulate neurite growth in an injury-dependent manner, we induced axonal damage *in vitro* by culturing neurons in the presence of camptothecin for 24 h. Camptothecin is an established neurotoxic agent inducing apoptosis and growth inhibition through DNA topoisomerase I inhibition and subsequent DNA damage accumulation [73]. We added NGF to the cultures to prevent complete neurite growth impairment and overt neuronal cell death.

Camptothecin administration resulted in a robust axon growth reduction in both wt (figure 9*e*) and ATF3-deficient (figure 9*f*) neurons. Addition of recombinant GRP enhanced neurite growth in wt neurons in the presence of camptothecin, however not in a statistically significant manner (figure 9*i*). In *Atf3* mutant neurons, GRP elevated neurite growth significantly in the presence of camptothecin. Now, neurite growth reached levels comparable to NGF treatment alone (figure 9*i*). Similar to GRP, VIP treatment elevated the camptothecin-mediated decrease in neurite growth of both wt (figure 9*g*) and ATF3-deficient (figure 9*h*) neurons. Administration of galanin or Wnt2b stimulated neurite growth of wt neurons in the presence of camptothecin. In contrast to this, both recombinant proteins had no effect on *Atf3* mutant neurons (figure 9*i*).

Thus, in primary PNS neurons, the neuropeptides GRP and VIP were able to rescue cell damage-associated axon growth in ATF3-lacking neurons.

### 3.7. ATF3 mediates repression of genes relevant to nerve injury and neurite growth

So far, we analysed genes whose expression was reduced in injured ATF3-deficient compared with wt FN (figure 9). However, in the initial transcriptomics approach (figures 6 and 7), selected genes such as *Timp1*, *Nov* or *Ccl2* were more upregulated in *Atf3* mutants upon nerve injury*. Timp1* (figure 7) and *Nov* (electronic supplementary material, figure S3*i*) expression profiles obtained in microarrays were confirmed by qPCR.

**Figure 10.**
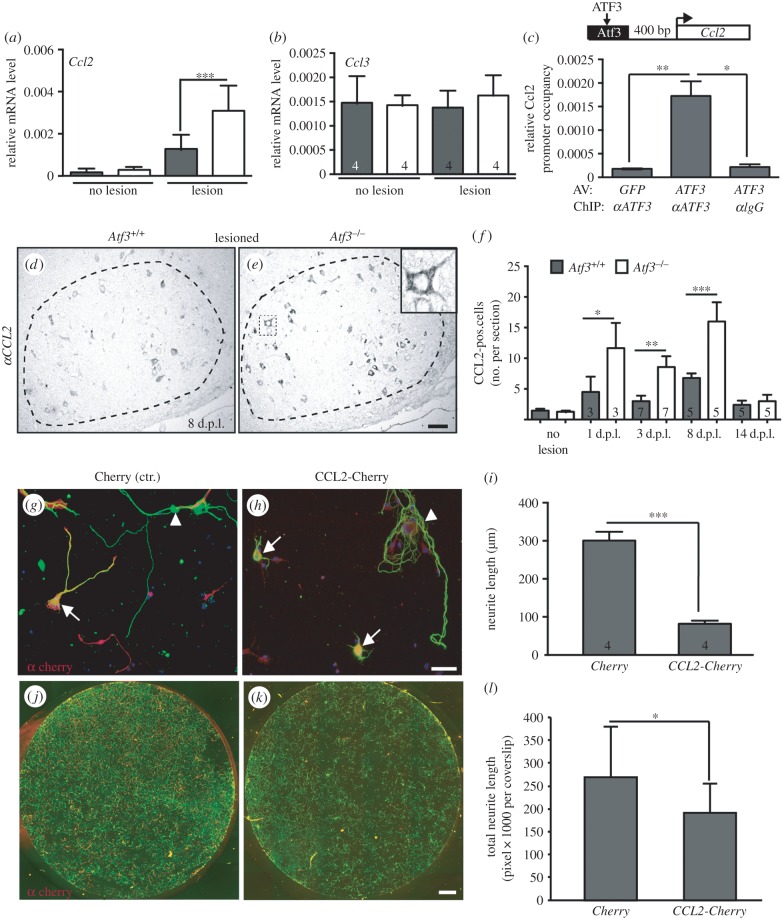
CCL2 is upregulated in *Atf3* mutants and CCL2 overexpression reduces neurite growth. (*a,b*) *Ccl2* (*a*) but not *Ccl3* (*b*) mRNA was more strongly induced in injured *Atf3* mutant FN compared with wt. (*c*) In ChIP experiments, ATF3 occupied ATF3 binding sites in the *Ccl2* promoter. (*d–f*) In lesioned wt FN, CCL2-directed antibodies labelled FMNs at 8 d.p.l. (*d*). The number of CCL2 positive FMNs was elevated in injured ATF3-deficient mice (*e*). Higher magnification (inset in (*e*)) suggested CCL2 localization in cytoplasmic vesicles. (*f*) Quantification of CCL2 positive neurons per section at different times post-injury. (*g–l*) Wt DRG neurons were electroporated with expression vectors driving Cherry expression alone (*g,j*) or CCL2-Cherry (*h,k*). Cherry positive neurons were identified with anti-Cherry (red) and anti-βIII tubulin (green) directed antibodies. CCl2-Cherry positive neurons (arrows in *h*) and cultures (*k*) have decreased neurite growth and neuronal network density compared with control condition (*g,j*). (*i,l*) Quantification of neurite length of individual Cherry or CCL2-Cherry positive neurons (*i*) or of all neurites present on the entire coverslip regardless of Cherry expression (*l*). Numbers in bars indicate numbers of independent experiments or animals analysed. Data are presented as mean ± s.d. *p ≤ 0.05; **p ≤ 0.01; ***p ≤ 0.001. Scale bar (*d,e*) = 100 µm; (*g,h*) = 50 µm; (*j,k*) = 1 µm.

In further analysis (figure 10), we focused on *Ccl2*, a gene encoding a secreted chemokine, also known as monocyte chemoattractant protein 1 (MCP-1), previously connected to PNS axon regeneration. PNS injury upregulates *Ccl2* in DRGs [27,74,75] and FMNs [76]. So far, CCL2 has been attributed functions during nerve injury-associated immune responses mainly by regulating monocyte rather than neuronal responses [23–29]. In this study, we focus on a potential neuronal response mediated by CCL2 (figure 10). First of all, we confirmed approximately twofold augmented *Ccl2* mRNA levels in axotomized FN of *Atf3* mutant mice compared with wt (figure 10*a*; figure 6*a*). Similarly elevated *Ccl2* levels were also observed in *Atf3* mutant mice in an ischaemia model [21]. This effect was specific for *Ccl2,* as other CCL chemokines, such as *Ccl3*, were unaffected (figure 10*b*). *Ccl2* upregulation upon ATF3 loss-of-function suggests repression of the *Ccl2* promoter by wt ATF3. To test whether this might be exerted by direct ATF3 binding to the *Ccl2* promoter in neurons, we employed ChIP with ATF3 binding sites reported in other cell types [22]. As seen for neuropeptides (figure 8), ATF3 also occupied potential ATF3 binding sites of the *Ccl2* promoter (figure 10*c*). Furthermore, the CCL2 protein abundance was investigated (figure 10*d–f*). In unlesioned FN of either genotype, anti-CCL2-directed antibodies did not recognize any positive cells (data not shown), a finding in line with mRNA levels (figure 10*a*). By contrast, in injured wt FN, several FMNs were CCL2 positive (figure 10*d*; quantified in *f*). Inspection of ATF3-deficient injured FN (figure 10*e*) revealed an increase of CCL2 positive FMNs at several timepoints post-injury (figure 10*f*).

Next, we analysed functional consequences of a neuronal CCL2 localization such as neurite growth modulation. So far, the impact of CCL2 on neurite growth has not been investigated in great detail. CCL2 induced primary neuronal cell death, suggesting a negative role on neurons [77]. A further study reported that CCL2 administration to the culture medium failed to enhance neurite growth whereas intra-thecal CCL2 delivery primed DRGs for enhanced growth *in vitro* [27]. To address consequences of elevated intracellular CCL2 levels as observed in ATF3-deficient neurons (figure 10*e*), we analysed the impact of elevated CCL2 levels in wt DRG neurons (figure 10*g–i*). For this, CCL2 was overexpressed within DRG neurons by electroporation of a CCL2–Cherry fusion construct rather than applying CCL2 to the medium reported to be ineffective [27].

In control electroporated DRG neurons, expressing Cherry alone, individual Cherry positive DRG neurons elaborated several neurites (arrow, figure 10*g*). In general, neurons were indistinguishable from Cherry negative neurons on the same coverslip (arrowhead, figure 10*g*). In contrast to this, overexpression of CCL2-Cherry decreased neurite growth, with most DRG neurons protruding only short neurites (arrows, figure 10*h*). Thus, elevation of neuron intrinsic CCL2 expression resulted in impaired PNS neurite growth. Following up on this, we wondered whether CCL2 expression might also affect neurite growth of surrounding CCL2 negative DRG neurons in a non-cell-autonomous manner (figure 10*j–l*). On control coverslips electroporated with Cherry alone (figure 10*j*), the overall neuronal network density was strongly increased compared with a CCL2-Cherry expressing culture (figure 10*k*; quantified in *l*). This finding suggests that CCL2 overexpression decreased neurite extension also by a paracrine mechanism.

In short, electroporation-mediated elevation of CCL2 levels in wt DRG neurons—as observed in *Atf3* mutant neurons—decreased primary PNS neurite growth.

### 3.8. ATF3 is upregulated in human peripheral nervous system injury

**Figure 11.**
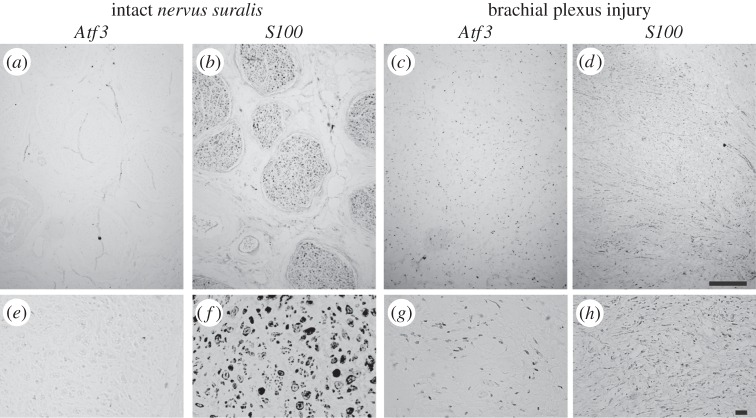
ATF3 is expressed in human peripheral nerve injury. (*a,b,e,f*) A non-injured human *nervus suralis* nerve was stained for ATF3 (*a,e*) and S100 (*b,f*). ATF3 was nearly absent from this control nerve sample (*a,e*). (*c,d,g,h*) A section of a C5 brachial plexus injury was stained for ATF3 (*c,g*) and S100 (*d,g*). ATF3 was expressed in this PNS injury sample and appeared to co-localize with several S100 positive Schwann cells labelled on a neighbouring section (compare (*g*) and (*h*)). Scale bar (*a–d*) = 500 µm; (*e–h*) = 20 µm.

So far, to the best of our knowledge, ATF3 expression was not investigated in human peripheral nerve injury. We tested whether ATF3 was also upregulated in human PNS injury, employing neuroma tissue derived from surgical resection of patients with brachial plexus injuries. As control for an intact nerve, we used freshly isolated *nervus suralis* sections of the same patient (figure 11).

In the *nervus suralis*, ATF3 was only weakly expressed (figure 11*a,e*). Neighbouring *nervus suralis* sections were stained for the Schwann cell marker S100 to show Schwann cells surrounding peripheral axons (figure 11*b,f*). By contrast, in a C5 brachial plexus lesion, ATF3 expression was found throughout the entire section (figure 11*c,d*). To address the cellular identity of ATF3 expressing cells, neighbouring sections were stained for S100 (figure 11*d,h*). As neuronal cell bodies are not present in these nerve samples, we reasoned that Schwann cells might be upregulating ATF3 upon injury. Indeed, ATF3 and S100 showed similar staining patterns indicating the presence of ATF3 in Schwann cells (compare figure 11*g,h*). However, not all S100 positive signals appeared to be ATF3 positive suggesting that not all Schwann cells did express ATF3.

Overall this finding points at ATF3 expression in human Schwann cells, similar to PNS injury in rodents (figure 1).

## 4. Discussion

### 4.1. The time course and localization of ATF3 expression upon peripheral nervous system nerve injury

ATF3 expression in FMNs peaked in the first week after injury (figure 1) and was nearly absent from 14 d.p.l. onwards, a time period when robust innervation of facial muscles by regenerating motor axons started (figure 3). This expression profile suggests that ATF3-regulated effector RAGs might predominantly influence early processes of FMN regeneration, such as initial outgrowth of motor axons starting two to three days after injury. In accordance with this observation was a reduction in facial nerve sprouting in ATF3-deficient animals at 3 d.p.l. (figure 4). Nevertheless, the mRNA expression profile of several injury-regulated genes points at a function of ATF3 also at later timepoints after injury. For instance, high mRNA levels of several injury-induced genes (e.g. *Galanin, Wnt2b. Ngf, Grp, Sprr2j*) were observed also at later injury timepoints, i.e. at 7 and sometimes even up to 14 days after facial nerve lesion (electronic supplementary material, figure S2). This suggests that gene products of such ATF3-regulated effector RAGs (figures 6 and 7) might still be present in the second week after injury and later. In accordance, we observed a high protein abundance of the ATF3-regulated RAG galanin in FMNs at 12 days after injury (figure 7*l*). Thus, at the protein level, ATF3-regulated gene products may influence also later regeneration processes such as long distance axon growth resulting in facial muscle re-innervation, although ATF3 itself is nearly absent. Besides FMNs, ATF3 induction was also observed in rodent (figure 1) and human (figure 11) Schwann cells after peripheral nerve injury. Ablation of the ATF3 partner protein c-Jun uncovered the importance of Schwann cells in PNS regeneration [51,52]. In our study, ATF3 was constitutively deleted from all cells. Thus, besides FMNs, impaired axon regeneration observed in *Atf3* mutant mice (figures 2 and 3) might also be a consequence of ATF3 depletion from Schwann cells.

### 4.2. ATF3's role in peripheral nervous system axon regeneration

In the unlesioned FN, topographic mapping and numbers of FMN subtypes were unchanged between adult wt and *Atf3* mutant mice (figure 2*n,o;r*). In agreement with ATF3's absence in the intact FN (figure 1*a*), this suggests no major role of ATF3 in topographic map formation and FMN generation during brain development and physiological FMN function. However, ATF3's function became obvious once FMNs were injured and FMN regeneration was analysed. Here, all three FMN subtypes analysed differed with regard to their regeneration potential. Thus, nearly 100% of DiI positive FMNs were re-connected to their eyelid target, whereas only 60% of FG and approximately 30% of Ctx488 positive FMNs reached their targets (figures 2 and 3). As the eyelid is closest to the initial lesion site, this outcome correlates with the distance axons had to bridge to reach their target (figure 2*a*(ii)). All three FMN subtypes differed also in numbers present in the FN and whiskers were represented by the largest FMN number (figure 2*n,o;r*).

Inspection of FMN regeneration to the whiskers revealed a significant reduction by approximately 30% in ATF3-deficient mice, indicating a necessary ATF3 contribution to PNS regeneration (figures 2 and 3). Nevertheless, although axon regeneration was slowed down in the absence of ATF3, it was not completely prevented. This points at additional factors contributing to complete nerve regeneration. In a current model of PNS axon regeneration, several TF-encoding RAGs form hubs within several hundred genes encompassing RAG networks. Recently, a detailed analysis of such RAG networks identified and as important TF hubs [8]. Given the presence of several TF hubs, single hub deletion as performed in this study might be compensated for by other hubs [5,6]. In accordance with this model, single deletion of other hub TFs such as *c-Jun* [13] or *p53* [16] reduced FMN regeneration to a similar extent as observed for *Atf3* mutant mice in this study. Thus, compound mutagenesis of hub TFs, e.g. *Atf3* and *c-Jun* double mouse mutants, might reveal stronger impairments in PNS regeneration compared with single mutants.

### 4.3. Identification of ATF3 target genes associated with peripheral nervous system axon regeneration

In this study, we provide a first genome-wide survey of ATF3 target genes employing ATF3 loss-of-function. Approximately 20–25% of the FMN RAG programme was to some extent ATF3-dependent (figure 6; electronic supplementary material, table S1), suggesting overall a modest impact of ATF3 on the total RAG response. However, individual genes or gene sets identified in this study showed a stronger ATF3 dependency. Specific RAGs including *Sprr2j and Wnt2b* and, particularly, a neuropeptide (*Pacap, Grp, Vip, Galanin, Ngf, Npy*) or neuropeptide receptor (*Vipr2, Avpr1a*) encoding gene cluster were strongly affected by ATF3 deficiency upon injury (figures 6–8). So far, transcriptional regulation of this gene cluster by ATF3 was not reported. Further analysis suggested indirect regulation of *Vip, Ngf, Pacap* and *Vipr2* by ATF3, whereas *Galanin* and *Grp* might be direct ATF3 target genes (figure 8). Laser microdissection experiments suggest that the RAG response primarily takes place in FMNs and not in other cell types (electronic supplementary material, figure S1).

Besides transcriptional activation, individual genes appeared to be under negative ATF3 transcriptional control. So far, gene repression by ATF3 is known in non-neuronal cells [18,78,79]. In this study, we identified genes whose expression is potentially repressed by ATF3 in neurons. For instance, *Timp1*, *Nov* and *Ccl2* were more upregulated in lesioned *Atf3* mutants suggesting gene repression by wild-type ATF3 (figures 6 and 10; electronic supplementary material, figure S3). *Ccl2* was also elevated in *Atf3* mutants during cerebral ischaemia [21] indicating a more general mechanism of *Ccl2* inhibition by ATF3.

### 4.4. ATF3-associated mechanisms of peripheral nervous system axon regeneration

**Figure 12.**
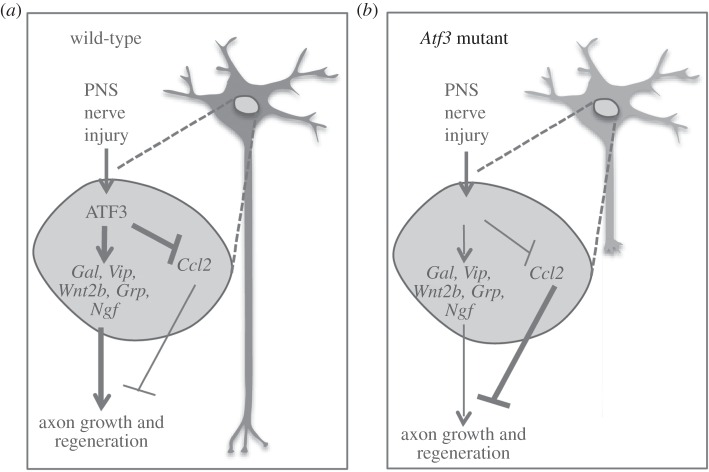
Summary of ATF3's function in facial nerve regeneration. (*a*) In injured wild-type mice, ATF3 is involved in transcriptional activation of the RAGs *Gal, Vip, Wnt2, Grp* and *Ngf*, whereas *Ccl2* is repressed. Neuropeptides would stimulate axonal growth and thereby increase axonal regeneration. By contrast, CCL2 levels in FMNs are suppressed by ATF3 and therefore CCL2's potential to decrease axon growth is reduced. (*b*) In lesioned *Atf3* mutant mice, induction of *Gal, Vip, Wnt2, Grp* and *Ngf* is reduced, resulting in weaker stimulation of axonal growth and also reduced regeneration potential of FMNs. In addition, *Ccl2* is not repressed by ATF3 anymore and enhanced CCL2 levels in injured FMNs decrease axonal growth and regeneration.

In this report, impaired facial nerve regeneration in *Atf3* mutants was associated with reduced induction of several RAGs including the neuropeptides *Galanin*, *Grp*, *Vip, Ngf* and *Wnt2*b as well as *Sprr2j*. Besides *Grp*, all the aforementioned genes or closely related family members can stimulate neurite growth and axon regeneration [63–68,80,81]. In addition, many neuropeptides affect neuronal survival, pain perception and neurotransmitter release [82,83]. We directly tested whether any of these ATF3-regulated gene products can rescue neurite growth reduced upon ATF3 loss-of-function *in vitro*. Indeed, addition of recombinant GRP, VIP and, most pronounced, NGF were able to rescue neurite growth impairment induced by ATF3 deficiency (figure 9). Although confirmation of such a rescue effect is missing upon facial nerve injury *in vivo*, these *in vitro* data highlight NGF, GRP and VIP as potential candidates responsible for ATF3's pro-regenerative function (see summary figure 12).

Data above suggest positive regulation of direct growth-promoting molecules by ATF3 upon nerve injury (see summary figure 12). In addition, we also observed a potential ATF3-mediated repression of genes such as *Ccl2* and *Nov* (figures 6 and 10; electronic supplementary material, table S1 and figure S3). Both *Ccl2* (figure 10) and *Nov* (electronic supplementary material, figure S3) were more strongly upregulated in injured *Atf3* mutant FN. In axon regeneration, CCL2 has beneficial functions on immune cells, e.g. during removal of myelin debris. However, CCL2 also triggers axonal damage in mouse models of motoneuron disease [23] and multiple sclerosis [25]. The latter finding is in agreement with our *in vitro* data, demonstrating neurite growth inhibition by CCL2 overexpression (figure 10). Similar to CCL2, *Nov* (CCN3) has also been attributed growth inhibitory functions [84]. Thus, a further mechanism by which ATF3 enhances axon regeneration might involve repression of axon growth-inhibiting molecules such as CCL2 and CCN3 (see summary figure 12). At first glance, this appears contradictory, because *Ccl2* and *Nov* were also induced in wild-type mice upon injury (figure 6 and electronic supplementary material, table S1). As CCL2 mediates an immune cell response after injury, certain CCL2 levels might be beneficial for axon regeneration. However, excess CCL2 levels, as observed in ATF3-deficient neurons or upon CCL2 overexpression in wt neurons (figure 10), might counteract the neuron intrinsic axon growth potential. Thus, ATF3 might play a role in balancing CCL2 levels for an optimal regeneration outcome (see summary, figure 12).

## 5. Conclusion

In this study, we provide a first description of an ATF3 pro-regenerative function employing *Atf3* mouse mutants. For this, ATF3 appears to induce axon growth-stimulating molecules such as neuropeptides (e.g. galanin, VIP, GRP and NGF) and downregulate growth-inhibiting molecules such as CCL2. ATF3 loss-of-function data in this study fit well with ATF3 gain-of-function results, showing enhanced axon growth and PNS regeneration in neurons or mice overexpressing ATF3 [8,11,12,30]. In general, ATF3's stimulatory role in regeneration is in accordance with other ATF3 functions such as providing neuroprotection in mouse epilepsy [32,85], ischaemia [34], neurotoxicity [33] or ALS [31] models. As ATF3 is induced by numerous types of neuronal injury in many cell types, our findings on ATF3-mediated mechanisms in PNS injury might also hold true in other neuronal injury conditions.

## Supplementary Material

Supp Figs 1-5

## Supplementary Material

Supp Table 1

## Data accessibility

The datasets supporting this article have been uploaded as part of the electronic supplementary material.
