# Supplementary material for: Atf3 mutant mice show reduced axon regeneration and impaired regeneration-associated gene induction after peripheral nerve injury
Source: Open Biol. 2016 Aug 31;6(8):160091. doi: 10.1098/rsob.160091 (PMC5008009; doi:10.1098/rsob.160091)
Supplement: Supp Figs 1-5 [file rsob160091supp1.pdf]

Supplementary information

Atf3 mutant mice show reduced axon regeneration and impaired regeneration associated gene (RAG) induction after peripheral nerve injury

Manuel Gey, Renate Wanner, Corinna Schilling, Maria T Pedro, Daniela Sinske and Bernd Knöll*

Institute of Physiological Chemistry

Ulm University

Albert-Einstein-Allee 11

89081 Ulm

Germany

Department of Neurosurgery

Bezirkskrankenhaus Günzburg

Ulm University

89081 Ulm

Germany

* to whom correspondence should be addressed:

bernd.knoell@uni-ulm.de

phone +49 731 500 33839

fax +49 731 500 2289

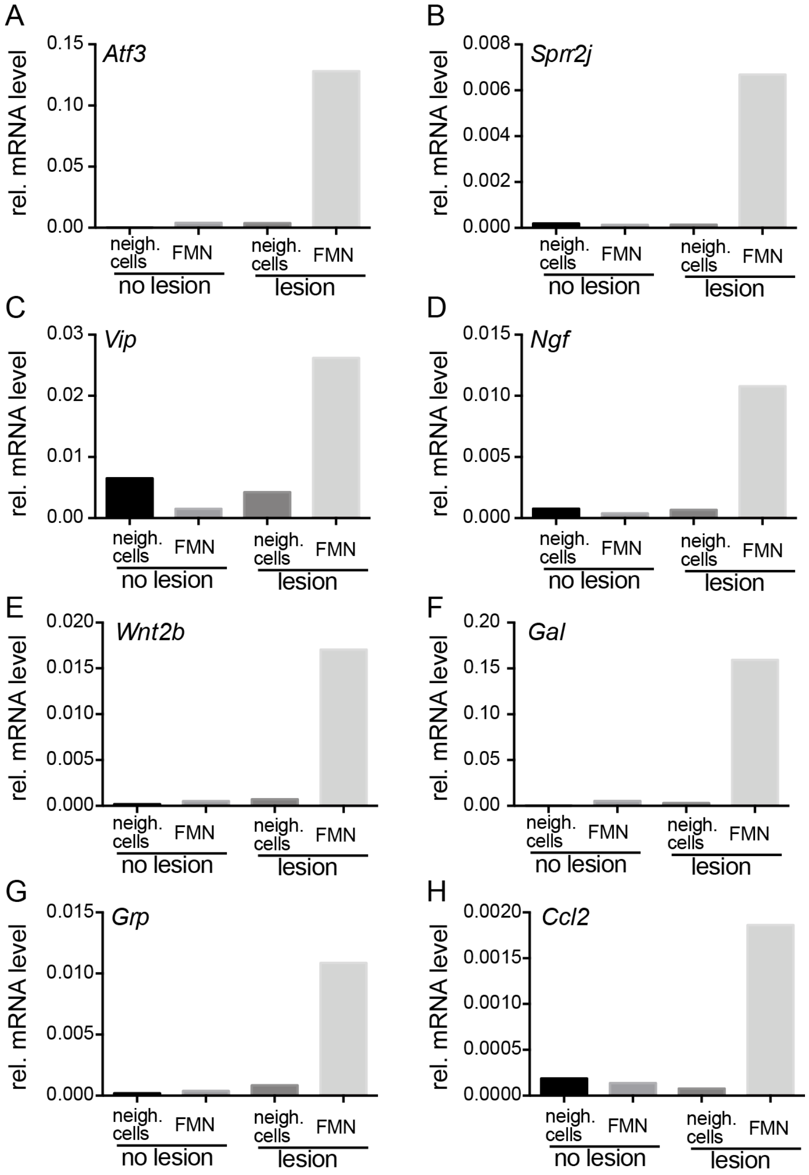

Supp. Fig. 1

Transcriptional changes during facial nerve injury mainly take place in FMNs

(A-H) Three days after facial nerve injury, FMNs in the unlesioned and lesioned side were isolated by laser microdissection. In addition, tissue including neighboring cells ("neigh. cells") but not FMNs was harvested from the unlesioned and lesioned side. Many sections were pooled to obtain sufficient cDNA amounts for qPCR analysis of genes indicated (N = 1). mRNA abundance of all eight genes inspected was strongest in the FMN sample derived from the lesioned side. In contrast all three other samples only contained low mRNA levels of indicated injury-regulated genes. This suggests that injury associated transcriptional changes mainly take place in FMNs on the lesioned side.

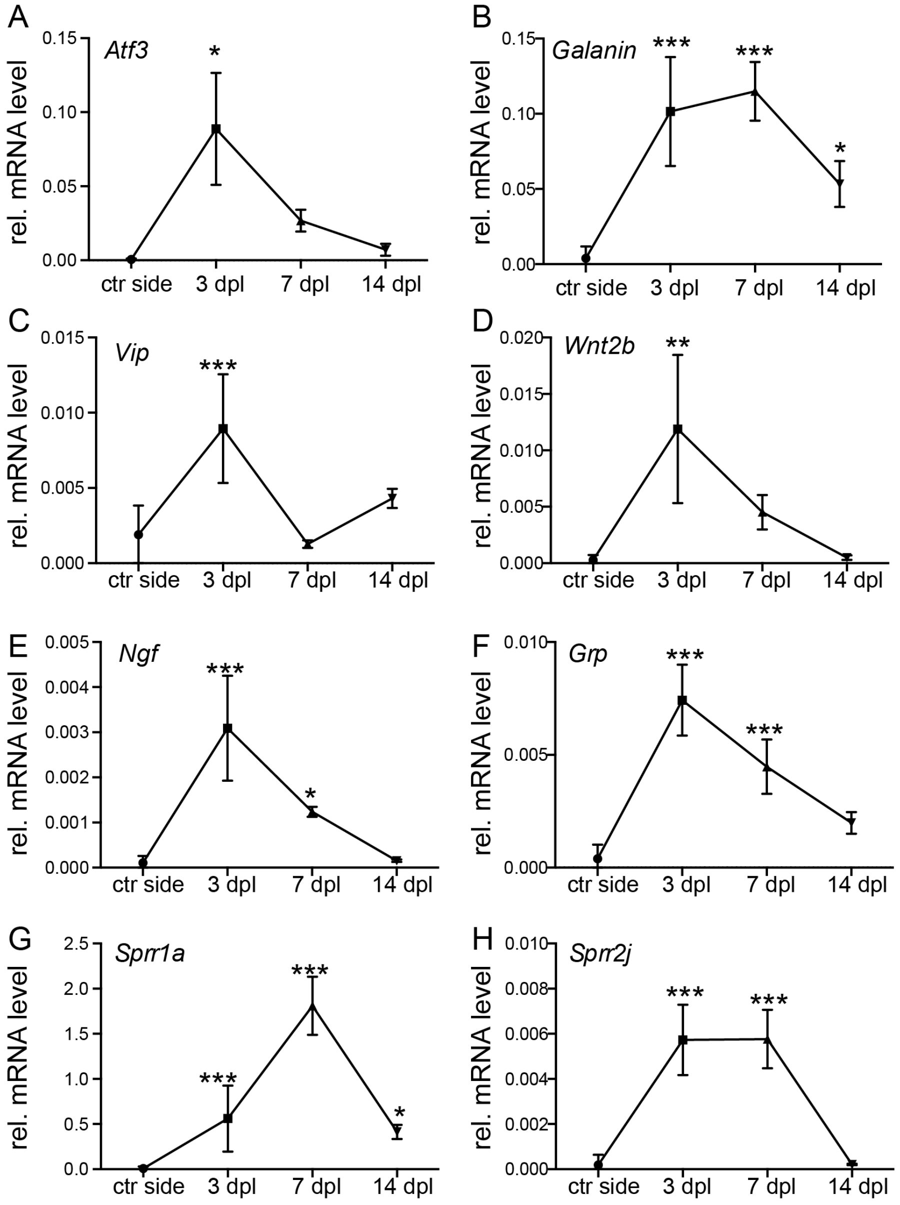

Supp. Fig. 2

Time course of injury-associated gene expression in the FN

(A-H) Unlesioned ("ctr side"; N = ≥ 8 mice) and lesioned FN at three (N ≥ 5 mice), seven (N = 3 mice) and 14 (N = 3 mice) days after facial nerve lesion were subjected to qPCR analysis for genes indicated. In general, maximal gene induction was achieved at three days post lesion. However, several genes including *Galanin, Ngf, Grp, Sprr1a* and *Sprr2j* were still significantly up-regulated at seven days post injury. Two weeks after injury, most genes returned to control level, the exception being *Galanin, Grp* and *Sprr1a*.

Data are presented as mean ± SD.

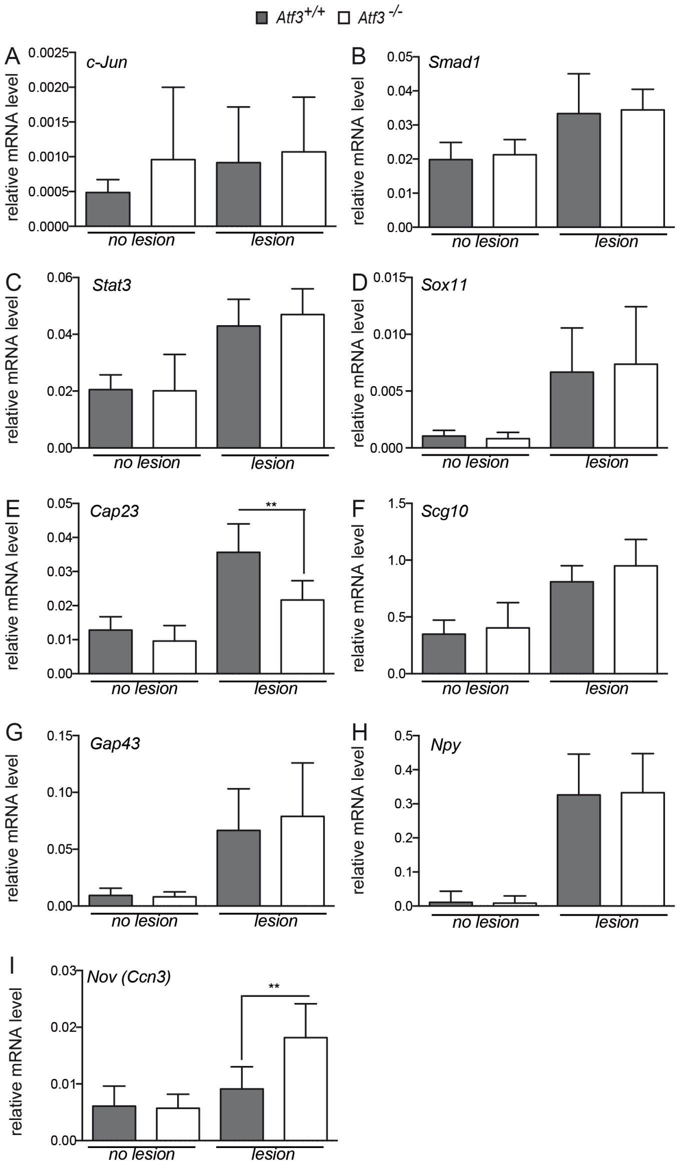

Supp. Fig. 3

Analysis of RAG expression in *Atf3* mutant mice

(A-H) Three days after facial nerve injury, mRNA was extracted from control (no lesion) and deafferented FN of wt (grey bars) and *Atf3* mutant (white bars) mice. All RAG genes analyzed by subsequent qPCR were induced in the FN upon facial nerve injury to a certain extent compared to the unlesioned samples. However, induction was comparable between wt and *Atf3* mutant mice suggesting that induction of all genes, with the exception of *Cap23* (E), is not dependent on ATF3. *Nov* expression was elevated in lesioned *Atf3* mutant FN compared to wt (I).

At least four independent experiments were performed for each bar.

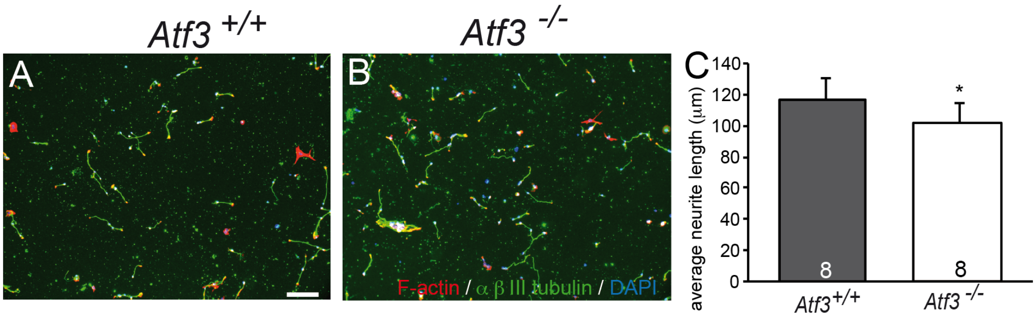

Supp. Fig. 4

Analysis of CNS neurite growth in *Atf3* mutant mice

(A, B) P3-P5 postnatal mouse cerebellar neurons derived from wt (A) or ATF3 deficient (B) animals were cultured for 48h. Neurite outgrowth was comparable between genotypes.

(C) Quantification of average neurite length from eight animal/genotype.

Scale-bar (A, B) = 100 μm

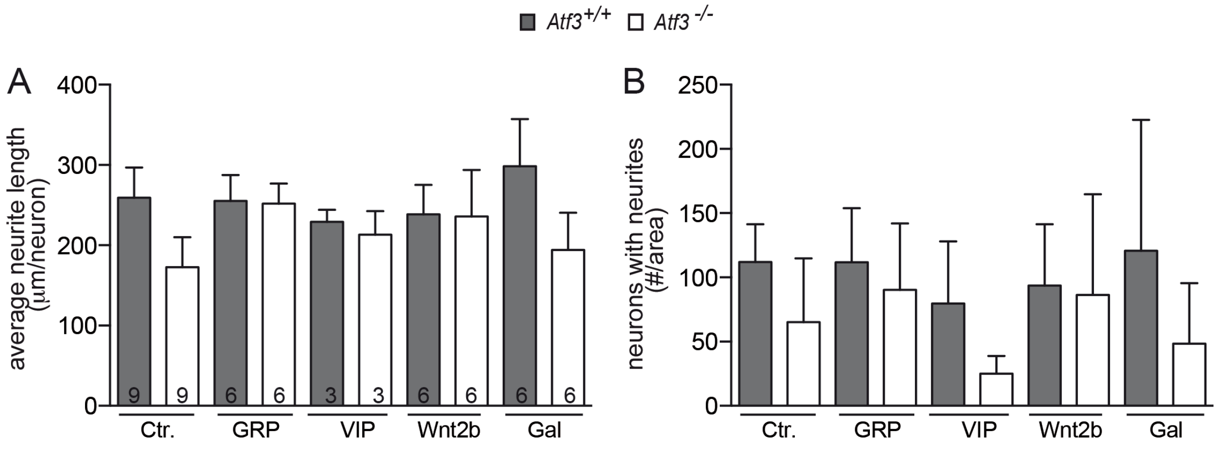

Supp. Fig. 5

Analysis of neuropeptides and Wnt2b in neurite growth

(A, B) Wildtype (grey bars) and *Atf3* mutant (white bars) adult mouse DRG neurons were cultured in the absence of NGF for 24h. GRP, VIP, Galanin or Wnt2b recombinant proteins were added to the culture medium as indicated. In (A) the average length of the longest neurites is quantified for each condition. In (B) the number of neurons with neurite growth/area is depicted. Numbers in bars indicate the numbers of independent animals tested for (A) and (B).

qPCR primer list

**Target Gene**	**Forward primer 5‘ → 3‘**	**Reverse primer 5‘ → 3‘**
Sprr2j	CTT GGC TGC ATC GCT TTG TA	TTG CAC TGC TGC TCT TGG TA
Vip	AAG AGG AGC AGT GAG GGA GA	TCA CGT GGT TGT TTT CCT TCG
Ngf	GGG AGC GCA TCG AGT TTT G	TAC GCT ATG CAC CTC ACT GC
Wnt2b	CGT GTA GAC ACG TCC TGG TG	GTA GCG TTG ACA CAA CTG CC
Gal	CAG TTT CTT GCA CCT TAA AGA GG	GGT CTC AGG ACT TCT CTA GGT CTT C
Grp	ACC GCT AAG TTG GTA GAC TCT C	CCC TTG TCG TTG TCC CTT CA
Timp1	GGC ATC CTC TTG TTG CTA TCA CT	CTT ATG ACC AGG TCC GAG TTG C
Hsp27	CTG GAC GTC AAC CAC TTC G	CTG CCT TTC TTC GTG CTT G
Vipr2	AGC AGT TCT ATA CTG CTT CCT GA	GGG CAC TTT CTG AGC CAT TC
Atf3	GCT GGA GTC AGT TAC CGT CAA	CGC CTC CTT TTC CTC TCA T
Atf3 overex.	GAT TAC AAG GAT GAC GAC GAT AG	CGA ACA CCA ATG ACC CAG GA
Ccl2	CCC AAT GAG TAG GCT GGA GA	TCT GGA CCC ATT CCT TCT TG
Ccl3	TGC CCT TGC TGT TCT TCT CT	GTG GAA TCT TCC GGC TGT AG
cJun	ACC CCC ACT CAG TTC TTG TG	AGT TGC TGA GGT TGG CGT AG
Smad1	GGT CTG CAT CAA CCC CTA CC	GAA CTG AGC CAG AAG GCT GT
Stat3	GGA AAT AAC GGT GAA GGT GCT	CAT GTC AAA CGT GAG CGA CT
Sox11	GAG CTG AGC GAG ATG ATC G	GAA CAC CAG GTC GGA GAA GT
Nov	CCG GGT CAC CAA CAG GAA TC	ATT TCT TGG TGC GGA GAC ACT
Scg10	CCA CCA TTG CCT AGT GAC CT	GAA GCA CAC ACT CCA CGA GA
Cap23	GGC GGC AGC GCT CCA ACT CG	CCG CCT GGG GTT CGC TCT CC
Npy	AGA AAA CGC CCC CAG AAC	GAT GAG GGT GGA AAC TTG GA
ChIP Primer		
Gal	GAT GAG GTA CTC GCC CCC TC	CTC ATC AGC AGT TCT CTG CTC C
Grp	ATG GGT CAA GGC ATA CTC AGC	GGG TTC TTA CAC CCG GGA C
Ccl2	TAA CCA CCA AGT GGA GAG AAT GCT G	GAA GTG GCC AAG GAA CCT AAA GTC
